# Maternal heterozygosity of *Slc6a19* causes metabolic perturbation and congenital NAD deficiency disorder in mice

**DOI:** 10.1242/dmm.049647

**Published:** 2022-11-14

**Authors:** Hartmut Cuny, Kayleigh Bozon, Rosemary B. Kirk, Delicia Z. Sheng, Stefan Bröer, Sally L. Dunwoodie

**Affiliations:** ^1^ Developmental and Stem Cell Biology Division, Victor Chang Cardiac Research Institute, Sydney, NSW 2010, Australia; ^2^ School of Clinical Medicine, Faculty of Medicine and Health, Sydney, NSW 2052, Australia; ^3^ Research School of Biology, Australian National University, Canberra, ACT 0200, Australia; ^4^ Faculty of Science, University of New South Wales, Sydney, NSW 2052, Australia

**Keywords:** Congenital malformation, Miscarriage, NAD, Metabolism, Embryonic development, Tryptophan

## Abstract

Nicotinamide adenine dinucleotide (NAD) is a key metabolite synthesised from vitamin B3 or tryptophan. Disruption of genes encoding NAD synthesis enzymes reduces NAD levels and causes congenital NAD deficiency disorder (CNDD), characterised by multiple congenital malformations*. SLC6A19* (encoding B^0^AT1, a neutral amino acid transporter), represents the main transporter for free tryptophan in the intestine and kidney. Here, we tested whether *Slc6a19* heterozygosity in mice limits the tryptophan available for NAD synthesis during pregnancy and causes adverse pregnancy outcomes. Pregnant *Slc6a19*^+/−^ mice were fed diets depleted of vitamin B3, so that tryptophan was the source of NAD during gestation. This perturbed the NAD metabolome in pregnant *Slc6a19^+/−^* females, resulting in reduced NAD levels and increased rates of embryo loss. Surviving embryos were small and exhibited specific combinations of CNDD-associated malformations. Our results show that genes not directly involved in NAD synthesis can affect NAD metabolism and cause CNDD. They also suggest that human female carriers of a *SLC6A19* loss-of-function allele might be susceptible to adverse pregnancy outcomes unless sufficient NAD precursor amounts are available during gestation.

This article has an associated First Person interview with the first author of the paper.

## Teaser

**Summary:** *Slc6a19*, encoding a neutral amino acid transporter, is the first non-enzymatic component of NAD metabolism shown to contribute to congenital NAD deficiency disorder, characterised by multiple malformations and embryo loss.

## INTRODUCTION

Major congenital malformations are a leading cause of death and disability in children worldwide. These malformations, defined as those with substantial medical consequences, occur in 3-6% of live births (Hobbs et al., 2014; Moorthie et al., 2018). Furthermore, 1-2% of women attempting to conceive experience recurrent miscarriage, defined as three consecutive miscarriages (Ford and Schust, 2009; Rai and Regan, 2006). A proportion of these miscarriages are attributable to abnormal embryonic growth or development (Khoury and Erickson, 1993; Philipp et al., 2003; Rai and Regan, 2006).

We recently showed that nicotinamide adenine dinucleotide (NAD) deficiency is associated with congenital malformation and miscarriage in humans (Shi et al., 2017; Szot et al., 2020, 2021). The malformations affect the heart, vertebrae and kidney, among other organs and tissues. In these studies, NAD levels were affected by biallelic mutation of *KYNU*, *HAAO* or *NADSYN1*. In mice, biallelic mutations in *Kynu or Haao* cause NAD deficiency, producing congenital malformations akin to those observed in human patients, as well as embryo loss (Cuny et al., 2020; Shi et al., 2017). *KYNU* encodes kynureninase, *HAAO* encodes 3-hydroxyanthranilic acid 3,4-dioxygenase, and *NADSYN1* encodes NAD synthetase 1. All three enzymes are required for NAD synthesis. NAD, referring to the redox couple NAD^+^ and NADH, is derived from dietary tryptophan through the NAD *de novo* synthesis pathway (also termed the kynurenine pathway), or from vitamin B3 via the Preiss-Handler and Salvage pathways (Dolle et al., 2013; Griffiths et al., 2020; Houtkooper et al., 2010). Vitamin B3 is a collective term for the NAD precursors nicotinic acid, nicotinamide and nicotinamide riboside. In mice, this genetic block in the conversion of tryptophan to NAD and subsequent NAD deficiency can be overcome by providing pregnant mice with sufficient vitamin B3 during gestation. Depending on the extent of vitamin B3 supplementation, adverse pregnancy outcomes can be reduced or completely prevented (Shi et al., 2017). Additionally, diets that are restricted in both NAD precursors, tryptophan and vitamin B3, cause NAD deficiency and multiple congenital malformations and miscarriage even in wild-type mice (Cuny et al., 2020). A less-restricted diet has the same effects when combined with a heterozygous loss-of-function mutation in *Haao*, demonstrating an interaction between the genetic and dietary components that yield NAD (Cuny et al., 2020). Although these studies show that genetic disruption of enzymes required to synthesise NAD from tryptophan can cause NAD deficiency during gestation, it is unclear whether disruption of non-enzymatic genes associated with tryptophan could affect NAD levels.

*SLC6A19*, encoding the neutral amino acid transporter B^0^AT1, mediates the uptake of neutral amino acids, and is the primary apical transporter of free tryptophan in the intestine and the proximal tubules of the kidney (Broer et al., 2011, 2004; Javed and Broer, 2019). Although tryptophan is an essential amino acid for protein synthesis, most of the new tryptophan entering the body via the diet is catabolised via different degradation pathways. The NAD *de novo* synthesis pathway is the major pathway in mammals, accounting for ∼90% of tryptophan catabolism. Thus, dietary supply of tryptophan is linked to NAD synthesis (Dolle et al., 2013; Sainio et al., 1996). Hartnup disorder, an autosomal-recessive disorder caused by mutations in *SLC6A19*, affects one in 30,000 individuals and is characterised by a severely elevated urinary neutral amino acid excretion (aminoaciduria) (Kleta et al., 2004; Scriver, 1965; Seow et al., 2004). In severe cases, patients develop Pellagra-like symptoms of low weight, dermatitis and neurological changes (Baron et al., 1956; Broer et al., 2005), and respond well to vitamin B3 (nicotinic acid) supplementation (Broer, 2009, 2005). *SLC6A19* loss-of-function mutant alleles have a prevalence of one per 578 in the Genome Aggregation Database (gnomAD v3.1.2) (Karczewski et al., 2020). Although heterozygous carriers are asymptomatic with respect to Hartnup disease, the implications of impaired tryptophan absorption on pregnancy have not been studied. Further studies are needed to address this, given that maternal plasma amino acid concentrations decline as foetal demand increases (Palou et al., 1977), and that limiting dietary tryptophan in mice causes congenital malformations and miscarriage as a result of gestational NAD deficiency (Cuny et al., 2020).

Here, we tested the impact of a heterozygous loss-of-function mutation in *Slc6a19* on the pregnancy outcomes in mice, when tryptophan is the dominant source of NAD during gestation. We aimed to determine whether pregnant *Slc6a19*^+/−^ mice have increased rates of dead and malformed embryos compared to pregnant wild-type mice. In addition, we aimed to investigate how the NAD metabolome of pregnant mice is affected by *Slc6a19* heterozygosity and whether any adverse effects on pregnancy result from NAD deficiency.

## RESULTS

### Maternal *Slc6a19^+/−^* genotype and NAD precursor-restricted diets during pregnancy cause loss of embryos and malformations in mice

We first aimed to determine whether mutations in *Slc6a19* can contribute to NAD deficiency and adverse pregnancy outcomes when combined with other non-genetic factors (gene–environment interaction). Pregnant *Slc6a19* heterozygous null (*Slc6a19^+/−^*) females were fed either standard diet or a NAD-precursor-restricted diet throughout gestation. Embryo survival and occurrence of malformations were assessed at embryonic day (E)18.5. The standard diet composition satisfies the nutritional requirements for growth of laboratory mice by providing 298 µg/day of NAD precursors and 10.53 mg/day of tryptophan (Cuny et al., 2020; Reeves et al., 1993). When converted to the human equivalents, it is above the recommended daily intake of NAD precursors (Cuny et al., 2020). In contrast, the diets with restricted amounts of NAD precursors, herein called restricted diets, have only 16-30% of the NAD precursor content of the standard diet (Table S1). The restricted diets are depleted in vitamin B3 to ensure that all NAD is derived from tryptophan itself via the NAD *de novo* synthesis pathway, with negligible input from the Preiss-Handler and Salvage pathways. Some of these diets were reported in a related study and caused NAD deficiency and adverse pregnancy outcomes in a dose-dependent manner in wild-type C57BL/6J and *Haao*^+/−^ mice (Cuny et al., 2020).

**Fig. 1.**
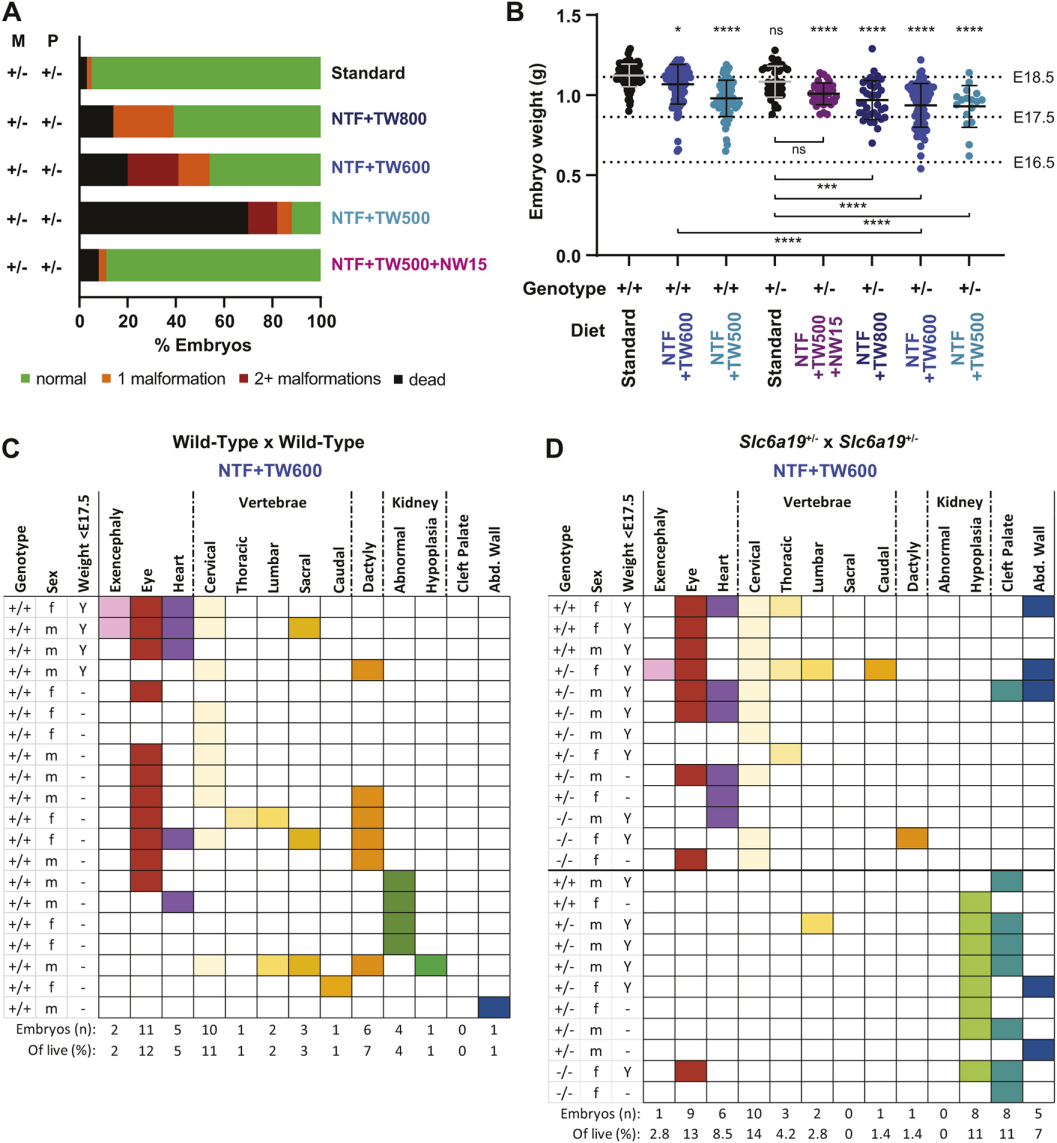
**Phenotypic outcomes in E18.5 mouse embryos of maternal *Slc6a19*^+/−^ genotype and diet.** (A) Summary of the phenotypic outcomes of embryos from *Slc6a19*^+/−^×*Slc6a19*^+/−^ matings. The maternal diet given throughout gestation was either not restricted in nicotinamide adenine dinucleotide (NAD) precursors (standard diet), reduced in NAD precursors to varying extents (NTF+TW500, NTF+TW600, NTF+TW800) or contained additional nicotinic acid in the drinking water (rescue condition, NTF+TW500+NW15). NTF, NAD precursor vitamin-depleted and tryptophan-free feed; NW, nicotinic acid-supplemented water; TW, tryptophan-supplemented water. See Table S1 for the specifics of the diets, Fig. S2 for the *Slc6a19*^+/−^ individual litter outcome data and Cuny et al. (2020) for the equivalent wild-type litter data. See Table 1 and Table S2 for numerical values and statistics. (B) Embryo weights at E18.5 for each of the different maternal diet and genotype combinations. Dissected embryos were weighed prior to assessment for congenital malformations. Bars indicate mean±s.d. Significance of differences relative to the wild-type standard diet group (first column) was determined by one-way ANOVA with Tukey's multiple comparisons test (****P<0.0001, ***P<0.001, *P<0.05; ns, not significant). See Table S3 for numerical values and statistics. Dotted lines indicate average weights of embryos from wild-type mothers fed the breeding diet and collected at E16.5, E17.5 and E18.5, respectively. (C,D) Illustration of the malformations present in E18.5 embryos of wild-type (C) or *Slc6a19*^+/−^ (D) mothers on the NTF+TW600 diet. Each row represents an embryo, and colours indicate the observed malformation(s). The three leftmost columns indicate, respectively, the embryos' genotype, sex and whether the embryo weighed less than an average E17.5 embryo from a wild-type mother on the breeding diet (Y, yes), as indicated by the dotted line in B. The numbers of embryos that had the indicated malformation and their percentage relative to all live embryos of the respective diet/genotype group are indicated at the bottom. The bold horizontal line in D arbitrarily separates the *Slc6a19*^+/−^ embryos into two subgroups based on their predominant malformation types, which are classified as ‘early’- and ‘late’-stage malformations. See Fig. 4 for a model linking the phenotypes of each subgroup to the presumed availability of NAD precursors. See Table S4 and Fig. S1 for details on the observed malformation types.

*Slc6a19*^+/−^ females were mated with *Slc6a19*^+/−^ males, resulting in embryos of all three genotypes, hereafter referred to as ‘*Slc6a19* litters’. Consistent with rates in litters of wild-type mothers, 92% of embryos from *Slc6a19*^+/−^ mothers fed the standard diet were alive, normal in weight and did not exhibit any gross morphological abnormalities, showing that the heterozygous maternal *Slc6a1*9 loss-of-function allele does not cause embryo loss or malformations when ample tryptophan and vitamin B3 are supplied (Table 1, Fig. 1A,B). However, tryptophan in this standard diet is provided as protein, enabling its intestinal absorption via both peptide transporters and B^0^AT1 (Broer, 2009; Daniel, 2004; Nassl et al., 2011). In contrast, our restricted diets provided all amino acids individually, increasing the dependency of tryptophan absorption on B^0^AT1. Additionally, pregnant females have a heightened requirement for dietary tryptophan for protein synthesis to meet the requirement for embryo growth and development (Palou et al., 1977).

**Table 1.**
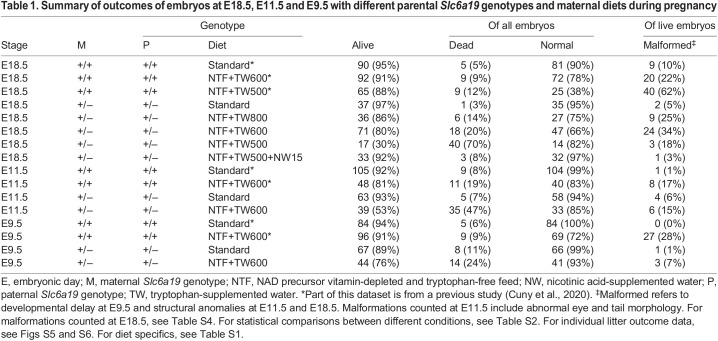
Summary of outcomes of embryos at E18.5, E11.5 and E9.5 with different parental *Slc6a19* genotypes and maternal diets during pregnancy

To determine whether there is sufficient tryptophan for normal development of embryos of *Slc6a19*^+/−^ mothers in our most restricted diet, pregnant *Slc6a19*^+/−^ mice were fed a combination of low tryptophan plus the vitamin B3 vitamer nicotinic acid [NAD precursor vitamin-depleted and tryptophan-free feed (NTF)+tryptophan-supplemented water (TW)500+nicotinic acid-supplemented water (NW)15, which provided 50.4% of the NAD precursor and 29% of the tryptophan supply of the standard diet] (Table S1). Observed pregnancy outcomes were comparable to those in *Slc6a19*^+/−^ mice provided with the standard diet (8% dead, 3% malformed) (Table 1, Fig. 1A; Table S2), indicating that tryptophan restriction itself does not result in malformations. Although these embryos weighed significantly less than embryos of wild-type mothers on the standard diet, there was no significant difference between their weight and that of embryos of *Slc6a19*^+/−^ mothers on the standard diet (Fig. 1B; Table S3). However, when the maternal dietary tryptophan supply was restricted in the absence of other NAD precursors, embryos from *Slc6a19*^+/−^ mothers incurred malformations associated with congenital NAD deficiency disorder (CNDD) (Cuny et al., 2020), such as structural heart, vertebral, kidney and eye defects (Fig. S1, Table S4). NTF+TW800 diet (47.1% of the tryptophan content of the standard diet and no added vitamin B3) resulted in 14% of embryos dead and 25% of live embryos malformed in *Slc6a19*^+/−^ litters. The NTF+TW600 diet (35.3% of the tryptophan content of the standard diet) increased the rate of embryo loss and malformation in surviving embryos in *Slc6a19^+/−^* litters (20% and 34%, respectively). The lowest tryptophan content diet, NTF+TW500 (29.4% of the standard diet), resulted in the death of 70% of embryos in *Slc6a19^+/−^* litters, with 18% of the remaining live embryos malformed (Table 1, Fig. 1A; Fig. S2). The percentage of embryo loss of *Slc6a19^+/−^* mothers on either restricted diet was significantly higher than that of wild-type mothers on the same diet (9% and 12%, respectively) (Table 1; Table S2).

Embryos from the *Slc6a19*^+/−^ litters fed all restricted diets weighed significantly less than embryos from *Slc6a19*^+/−^ mothers fed the standard diet (Fig. 1B; Table S3). To determine how small embryos of *Slc6a19*^+/−^ mothers were for their gestational age, we measured the weight of embryos from wild-type mice fed the breeding diet at E16.5, E17.5 and E18.5, respectively (average weights 0.58 g, 0.86 g and 1.13 g, respectively). Embryos at E18.5 that weighed less than the average weight of E17.5 embryos are defined here as small for their gestation age (Fig. 1B). The majority (67%) of malformed embryos from litters of *Slc6a19*^+/−^ mothers on NTF+TW600 were small for their gestational age, compared to only 20% of litters from wild-type mothers. The smaller embryo size was not linked to a specific sex because, among the affected embryos, one was female and three were male in litters from wild-type mothers, and seven were female and nine were male in litters from *Slc6a19*^+/−^ mothers (Fig. 1C,D).

Together, these results show that *Slc6a19*^+/−^ mothers can have normal pregnancies, even on a diet with strongly reduced tryptophan supply, if vitamin B3 (nicotinic acid) is also provided. However, on the restricted diets, *Slc6a19*^+/−^ litters have worse pregnancy outcomes than wild-type litters, evidenced by a higher incidence of dead embryos, indicating a gene–environment effect on embryogenesis.

### Maternal, but not embryo, *Slc6a19* genotype affects pregnancy outcome

Embryos of *Slc6a19*^+/−^ parents were more severely affected than those of wild-type parents. We investigated whether this difference was due to an increased death and malformation rate of *Slc6a19*^−/−^ embryos. It was not possible to genotype the resorbed embryos, but the surviving embryos adhered to Mendelian ratios across all dietary conditions. The proportion of malformed embryos and embryo weights were not significantly different among *Slc6a19^+/+^*, *Slc6a19^+/−^* and *Slc6a19^−/−^* embryo genotypes (Fig. S3). Similar types of malformations, in isolation or in combinations, were seen across the three genotypes (Fig. 1D for NTF+TW600). Therefore, embryos are not reliant on their own expression of *Slc6a19* for normal embryonic development, irrespective of how much tryptophan is provided.

### Maternal *Slc6a19*^+/−^ genotype combined with restricted diets affect embryo growth and occurrence of specific malformation

Of the NAD precursor-restricted diets tested, the NTF+TW600 diet produced the most malformed embryos in *Slc6a19*^+/−^ litters. Therefore, this diet was chosen in subsequent experiments and is referred to as the ‘restricted diet’ from here onwards. Although all malformations found in embryos of *Slc6a19*^+/−^ parents on the restricted diet were characteristic of NAD deficiency (Table S4, Fig. S1), the prevalence of some specific malformations differed compared to that in wild-type litters. To better understand the phenotypic differences between *Slc6a19*^+/−^ and wild-type litters and their causes, we examined the co-occurrence of individual malformations for each affected embryo.

In both wild-type and *Slc6a19*^+/−^ litters, heart, eye and vertebral defects tended to co-occur with a similar incidence (Fig. 1C,D). For wild-type litters, malformations such as polydactyly and abnormal kidney shape were also found in multiple embryos, but these malformations were not prevalent in *Slc6a19*^+/−^ litters (Table S4). Instead, a distinct subgroup of embryos from *Slc6a19*^+/−^ mothers exhibited a high incidence of kidney hypoplasia and cleft palate but very few other malformations (Fig. 1D). These kidney and palate malformations were virtually absent in embryos of wild-type mothers (Fig. 1C). Embryo size did not coincide with specific malformation types, because similar proportions of malformed embryos of *Slc6a19*^+/−^ mothers were classified as small for their gestational age in this additional subgroup (55%, 6/11), as well as in the subgroup with malformations akin to those of embryos of wild-type mothers (76%, 16/21). This suggests that embryo growth restriction and malformation might be caused by different mechanisms. Although placental defects have generally been linked to congenital malformation (Perez-Garcia et al., 2018) and growth restriction (Woods et al., 2018), the placentas of malformed or small embryos at E18.5 did not exhibit any obvious difference in gross structure or vascularisation beyond normal variability (Fig. S4). Similarly, the embryos' sex did not influence the incidence of specific malformations, because five embryos were females and six were males in the subgroup of embryos from *Slc6a19*^+/−^ mothers with frequent kidney and palate defects (Fig. 1D).

In summary, the E18.5 phenotyping data suggest that there are inherent differences in the timing and/or mechanisms causing malformations depending on maternal *Slc6a19* heterozygosity. This prompted us to assess the embryo phenotypes and embryonic NAD concentrations at earlier stages of embryogenesis.

### Maternal genotype and restricted diets affect early embryogenesis

We have previously shown that restricted diets reduce maternal and embryonic NAD levels during critical organogenesis timepoints E9.5 and E11.5, causing death and malformation (Cuny et al., 2020). Therefore, we assessed the survival and morphology of embryos at these key stages of embryogenesis. Because NAD deficiency causes malformations in organs that are usually used to stage embryos during mid-gestation (somites/vertebrae and limbs), the developmental stage was only assessed at E9.5 prior to the occurrence of these defects. Mouse embryos turn by E9, i.e. they undergo an axial rotation and convert from a dorsally concave and ventrally convex morphology to a dorsally convex and ventrally concave appearance (Theiler, 1989). Hence, if embryos at E9.5 phenotypically resembled E9 or even younger embryos, determined by axial rotation, presence of a forelimb bud and overall size, they were deemed developmentally delayed. In contrast, visible external malformations were only observable and counted at E11.5. The numbers of dead and live embryos were counted at both stages.

As observed at E18.5, the restricted diet significantly increased embryonic death in *Slc6a19*^+/−^ mothers compared to wild-type mothers at both E9.5 and E11.5 (Table 1; Table S2, Fig. S5, Fig. S6). At E9.5, embryos of wild-type mothers on the restricted diet more frequently resembled earlier-stage embryos than those from *Slc6a19*^+/−^ mothers. But, despite this early delay, most embryos of wild-type mothers on this diet were normal in size and morphology at E18.5 (Fig. 1B), suggesting that the early delay has no observed lasting impact on development. Taken together, these results indicated that the restricted diet drives developmental delay in wild-type litters, specifically at E9.5, but delay is absent at later timepoints. In contrast, this diet causes increased death in *Slc6a19^+/−^* litters at all embryonic timepoints.

### Extent of embryonic NAD deficiency depends on maternal, not embryonic, genotype

**Fig. 2.**
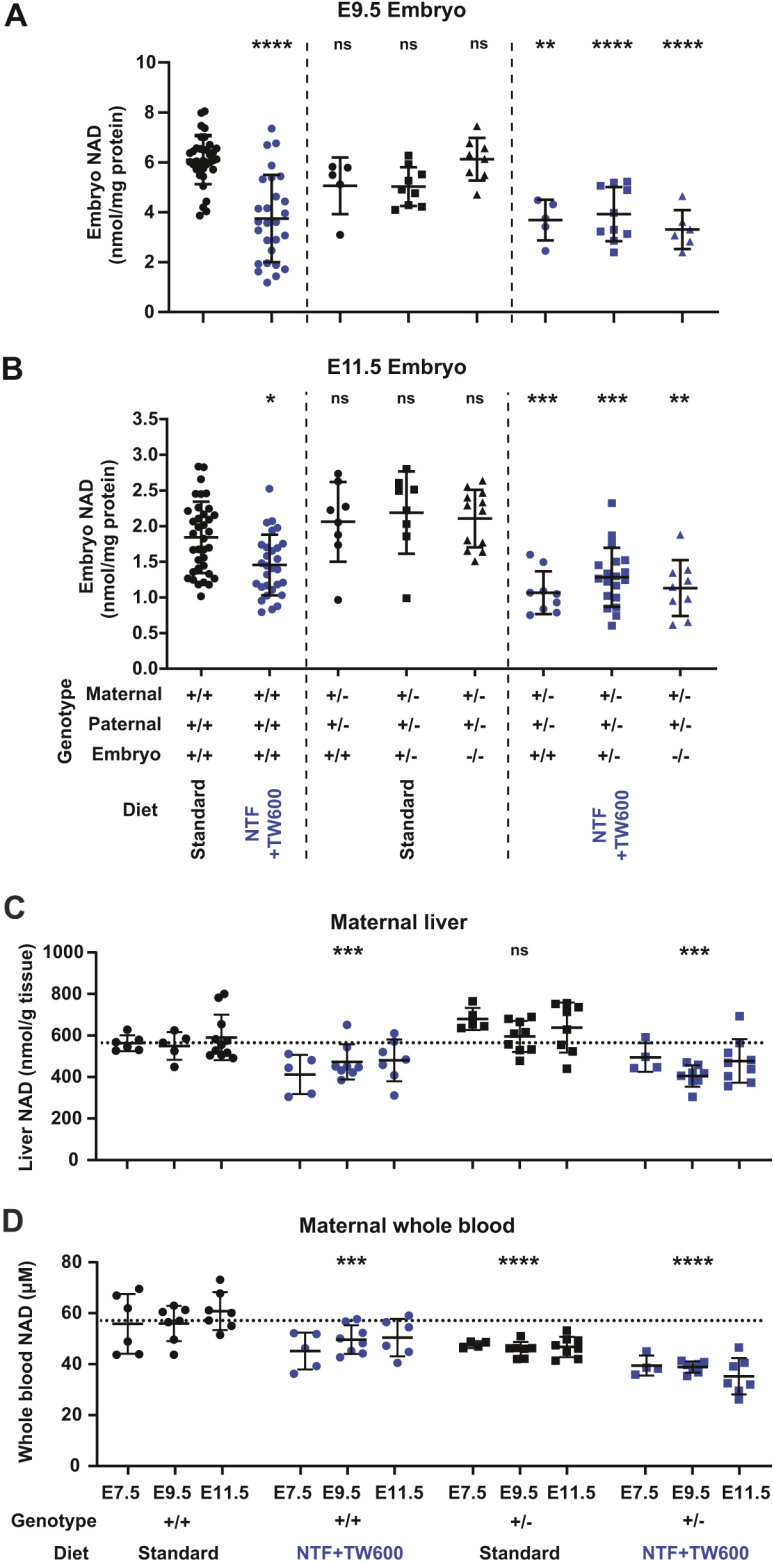
**Effects of *Slc6a19* genotype, diet, and gestational stage on embryo and maternal NAD concentrations.** (A,B) Total NAD (NAD^+^ and NADH) concentrations were measured in whole embryos at E9.5 (A) and E11.5 (B). Dots represent NAD concentrations of individual embryos, and bars indicate the mean±s.d. The parental and embryo *Slc6a19* genotypes and dietary conditions during pregnancy are indicated at the bottom. (C,D) Maternal liver (C) and whole-blood (D) total NAD (NAD^+^ and NADH) concentrations of pregnant mice under different genetic and dietary conditions, collected at different stages of gestation. Dots represent NAD concentrations in individual mice, and bars indicate the mean±s.d. The *Slc6a19* genotype, dietary condition during pregnancy and gestational stage at sample collection are indicated at the bottom. Maternal samples were collected, along with the embryos and deciduae for which NAD levels are summarised in A, B and Fig. S7. Dotted lines in C and D indicate the average NAD concentration of the wild-type standard diet control group (average of first three columns; black circles). Significance of differences relative to the wild-type standard diet group (first column in the embryo NAD graphs and average of first three columns in maternal NAD graphs) was assessed by one-way ANOVA with Tukey's multiple comparisons test (****P<0.0001, ***P<0.001, **P<0.01, *P<0.05; ns, not significant). See Tables 2 and 3 for numerical values and statistics.

We measured total NAD (NAD^+^ and NADH) levels in embryos at E9.5 and E11.5 to confirm that the adverse phenotypic outcomes in *Slc6a19*^+/−^ litters were caused by NAD deficiency. The restricted diet significantly reduced NAD levels in all embryos of *Slc6a19^+/−^* mothers at E9.5 and E11.5, irrespective of the embryo genotype (Fig. 2, Table 2; Table S5). This agrees with the E18.5 data, showing similar proportions of malformed embryos across all three genotypes (Fig. S4). Maternal genotype did not exacerbate the effect of the restricted diet on embryo NAD levels at E9.5, but, at E11.5, there was a small, albeit not statistically significant, reduction in NAD levels in embryos from *Slc6a19*^+/−^ mothers compared to those from wild-type mothers (*P*=0.0991, Table S5). Failure to reach significance could, in part, be due to the high variability in embryo NAD levels from litter to litter (Fig. 2). Furthermore, this variability in embryo NAD levels itself may explain the heterogeneity in severity and phenotypic outcomes observed at E18.5 (Fig. 1D; Fig. S2), E11.5 (Fig. S6) and E9.5 (Fig. S5).

**Table 2.**
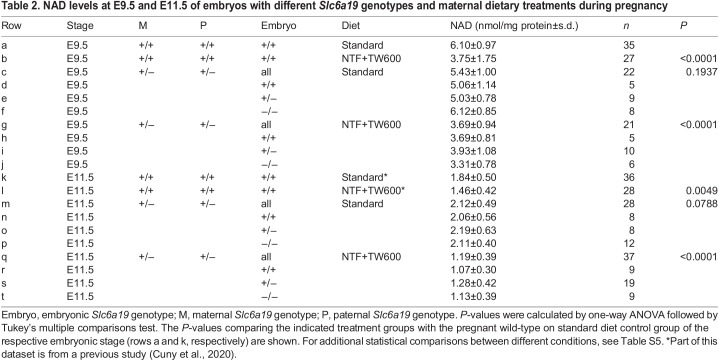
NAD levels at E9.5 and E11.5 of embryos with different *Slc6a19* genotypes and maternal dietary treatments during pregnancy

**Table 3.**
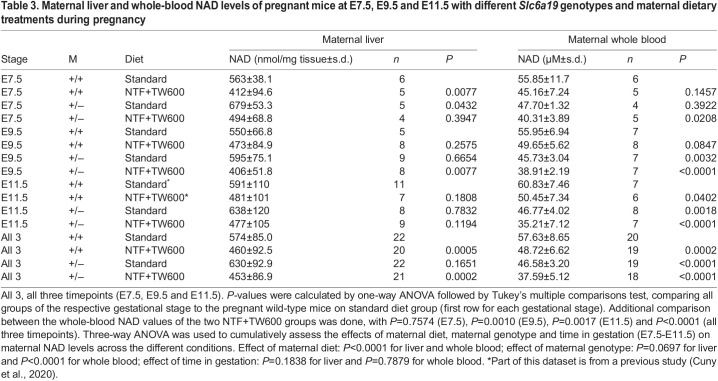
Maternal liver and whole-blood NAD levels of pregnant mice at E7.5, E9.5 and E11.5 with different *Slc6a19* genotypes and maternal dietary treatments during pregnancy

### Maternal genotype and restricted diets affect maternal NAD levels

It is likely that the maternal NAD levels reflect the availability of tryptophan and nicotinamide to the embryo. Therefore, to better understand how the *Slc6a19* genotype and NAD-precursor-restricted diet affect maternal NAD levels, we measured NAD in the maternal liver and whole blood collected at E7.5, E9.5 and E11.5 (Fig. 2C,D). Three-way ANOVA was performed for all gene–environment conditions and timepoints to compare the effects of maternal genotype, diet and stage of gestation on maternal NAD levels. As expected, diet significantly affected NAD levels in both the liver and whole blood. Maternal genotype did not significantly contribute to the overall variation of liver NAD, but it did for whole-blood NAD. However, gestational time from E7.5 to E11.5 did not significantly affect liver or whole-blood NAD levels in any of the genotype/diet conditions (Table 3, Fig. 2). Therefore, we combined all three timepoints and assessed the impact of genotype and diet on maternal NAD levels individually.

Maternal *Slc6a19*^+/−^ genotype alone (standard diet) slightly increased liver NAD and significantly decreased whole-blood NAD (Table 3). This shows that, although *Slc6a19*^+/−^ females can maintain normal pregnancies, there are inherent and fundamental differences in organ NAD levels dependent on maternal genotype. The restricted diet alone (wild-type mothers on NTF+TW600) caused a significant decline in both liver and whole-blood NAD levels. Despite the differences in NAD precursor provision, whole-blood NAD concentrations were similar between *Slc6a19*^+/−^ mothers on standard diet and wild-type mothers on the restricted diet. Furthermore, genotype and diet combined (*Slc6a19*^+/−^ mothers on NTF+TW600), significantly reduced the whole-blood NAD levels compared to either factor alone. In contrast, liver NAD levels on the restricted diet were similar irrespective of genotype (Table 3, Fig. 2). To understand how maternal NAD levels might affect the developing embryo, we also measured total NAD levels in the deciduae of pregnant mice at E7.5. These contain the embryo tissues but are predominantly maternal tissue, which is in direct contact with the embryo itself. Like in whole blood, at E7.5, NAD levels were lowered in whole deciduae of *Slc6a19*^+/−^ mothers, even on the standard diet (Table S6, Fig. S7).

Together, the maternal NAD measurements show that liver, whole blood and decidua NAD levels are dictated by both genetic and environmental factors. The restricted diet results in decreased tissue NAD concentrations, likely because of reduced NAD precursor availability for NAD synthesis. This effect is exacerbated by *Slc6a19* heterozygosity, with a larger decrease in liver NAD relative to precursor-rich diets, and the lowest observed whole-blood NAD levels. Furthermore, the higher liver NAD levels, and lower whole-blood and decidua NAD levels, in *Slc6a19*^+/−^ mothers on the standard diet compared to those in wild-type mothers confirm that there are innate metabolic differences between the genotypes and suggest that metabolomic adaptation to *Slc6a19* heterozygosity occurs.

### Maternal *Slc6a19*^+/−^ genotype perturbs the circulatory NAD metabolome and exacerbates dietary effects

**Fig. 3.**
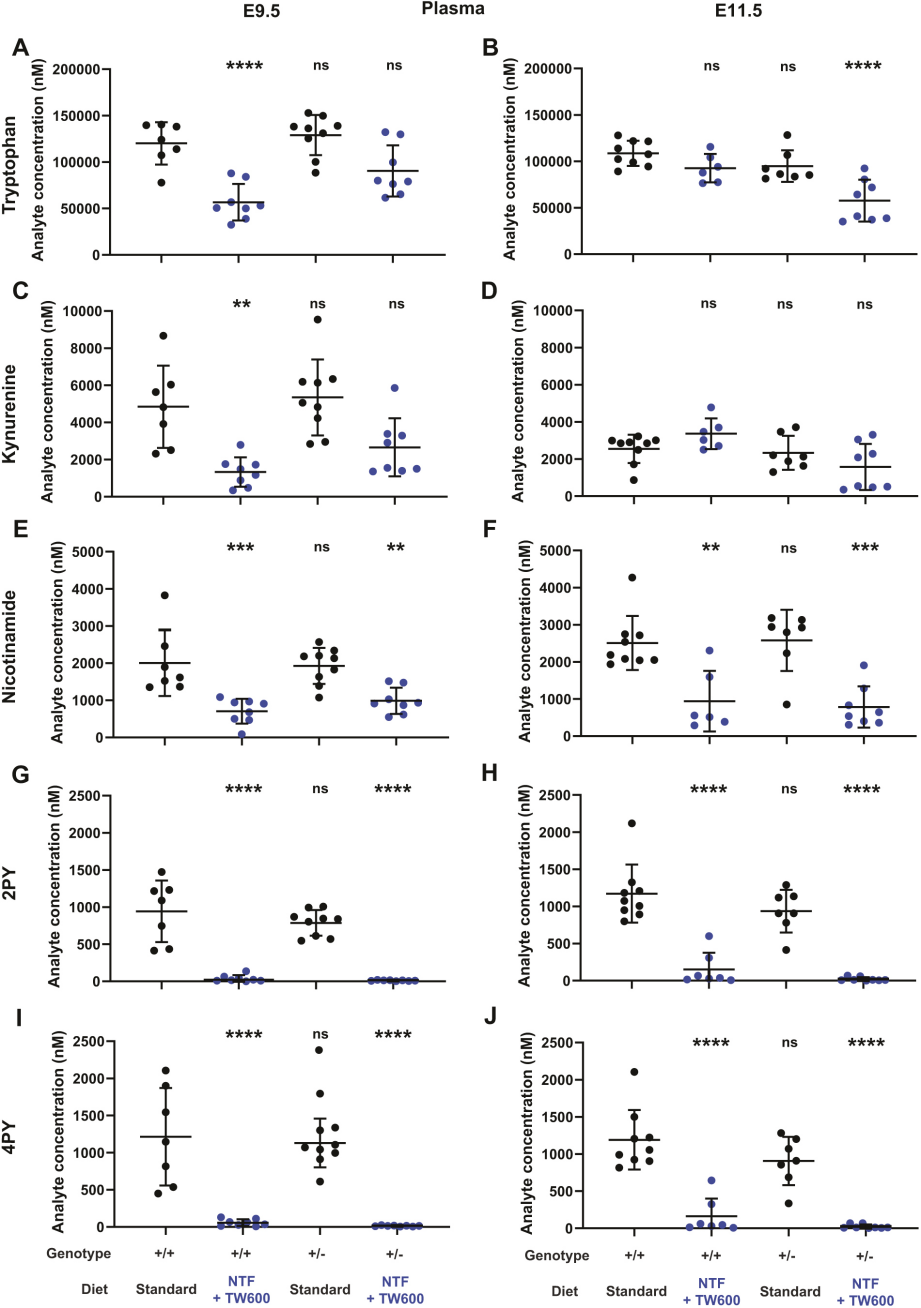
**Tryptophan, kynurenine, nicotinamide, 2PY and 4PY concentrations in plasma of pregnant wild-type and *Slc6a19*^+/−^ mice, measured by UHPLC-MS/MS.** (A-J) Plasma metabolite concentrations at E9.5 (A,C,E,G,I) and E11.5 (B,D,F,H,J). Dots represent metabolite concentrations in individual mice, and bars indicate the mean±s.d. The maternal *Slc6a19* genotype and diet are indicated at the bottom. Significance of differences in metabolite concentration relative to the pregnant wild-type standard diet group (first column) was assessed by one-way ANOVA with Tukey's multiple comparisons test (****P<0.0001, ***P<0.001, **P<0.01; ns, not significant). See Table 4 for numerical values and statistics. 2PY, N-methyl-2-pyridone-5-carboxamide; 4PY, N-methyl-4-pyridone-5-carboxamide.

Next, we investigated whether there are innate differences in the plasma NAD metabolomes of *Slc6a19*^+/−^ and wild-type mothers at E9.5 and E11.5 using an ultra-high-performance liquid chromatography–tandem mass spectrometry (UHPLC-MS/MS) method recently developed by us (Cuny et al., 2021). *Slc6a19*^+/−^ mothers on standard diet showed a non-significant decline in tryptophan concentration at E11.5. Restricted diet alone in wild-type mothers caused a significant decrease in tryptophan levels at E9.5, but they were largely restored at E11.5 and similar to those in *Slc6a19*^+/−^ mothers on the standard diet. This was reversed in *Slc6a19* heterozygotes on the restricted diet, which had significantly lower tryptophan concentrations at E11.5, but not at E9.5 (Fig. 3, Table 4). Kynurenine, the most abundant plasma metabolite downstream of tryptophan, generally followed the same trend as tryptophan based on maternal genotype and diet. Dietary NAD precursor restriction alone caused a significant drop in kynurenine levels at E9.5 (27.5% of those in wild-type mothers on standard diet), but this was restored completely by E11.5. However, when dietary restriction was combined with *Slc6a19* heterozygosity, kynurenine levels were lowest at E11.5 (62.0% of those in wild-type mothers on standard diet) (Fig. 3, Table 4). We assessed the effects of litter phenotype on tryptophan and kynurenine levels using two-way ANOVA. At E11.5, concentrations of tryptophan and kynurenine correlated with phenotype, whereby mothers with live litters had the highest concentrations of these metabolites, and those with dead litters had the lowest (Fig. S8).

**Table 4.**
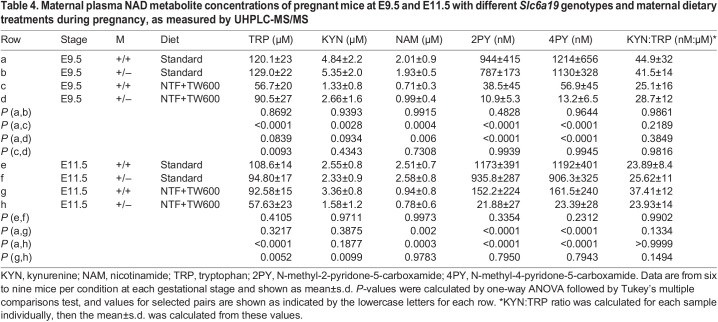
Maternal plasma NAD metabolite concentrations of pregnant mice at E9.5 and E11.5 with different *Slc6a19* genotypes and maternal dietary treatments during pregnancy, as measured by UHPLC-MS/MS

Tryptophan conversion to kynurenine via extrahepatic enzymes indoleamine 2,3-dioxygenase 1 and 2 (IDO1 and IDO2) is critical for immune tolerance, especially during pregnancy (Xu et al., 2017). Although elevated kynurenine to tryptophan (KYN:TRP) ratios are not exclusively determined by IDO1 and IDO2 induction, relative enzyme activity is commonly derived from this ratio (Schröcksnadel et al., 2006; Wang et al., 2015). Therefore, we determined how the differential availability of tryptophan affected these ratios. Despite the decline in tryptophan concentration under the restricted diet, KYN:TRP ratios were not significantly different from those under standard diet (Table 4; Fig. S9).

Nicotinamide is the main circulating NAD precursor exported from the liver for use in other organs (Liu et al., 2018). In contrast to tryptophan and kynurenine, the restricted diet was the only variable to impact circulating nicotinamide concentrations, with levels reaching only 32-40% of those in mothers on the standard diet at both gestational stages (Fig. 3, Table 4). The metabolites N-methyl-2-pyridone-5-carboxamide (2PY) and N-methyl-4-pyridone-5-carboxamide (4PY) are end products of NAD degradation, and their levels are influenced by the availability of nicotinamide (Shibata and Matsuo, 1989; Trammell et al., 2016). Regardless of genotype, 2PY and 4PY were both significantly reduced 25- to 92-fold in the restricted diet compared to standard diet (Fig. 3, Table 4).

Although plasma metabolite levels best reflect NAD precursor availability to embryos at a given gestational timepoint, their quantification is not always feasible, as it requires larger quantities of blood, and levels of some NAD-related metabolites are below the limit of quantification. To assess whether whole-blood samples can be used as a proxy for plasma, we quantified tryptophan, kynurenine, nicotinamide, nicotinamide mononucleotide (NMN) and NAD^+^ concentrations in blood of the same mice that yielded the plasma metabolite data. Generally, trends of metabolite levels under the genotype/diet conditions were similar to those of plasma (Table S7, Figs S10, S11 and S12), and whole-blood NAD^+^ and NMN concentrations of individual mice correlated with the survival of their litters (Figs S13 and S14).

In summary, the NAD metabolome is severely affected by dietary NAD precursor restriction. The timing and severity of these changes differs between the *Slc6a19*^+/−^ and wild-type mothers, with wild-type litters affected earlier in gestation.

## DISCUSSION

Embryo survival and normal development depend on provision of sufficient NAD levels during pregnancy. Reduced NAD levels during gestation due to loss-of-function mutations in NAD *de novo* synthesis pathway genes, severe dietary restriction of the NAD precursors tryptophan and vitamin B3, or a combination of both (i.e. gene–environment interactions) cause CNDD (Cuny et al., 2020; Shi et al., 2017; Szot et al., 2020). Although not an enzymatic component of the NAD synthesis pathway, the neutral amino acid transporter B^0^AT1 (encoded by *SLC6A19*) is crucial for tryptophan absorption in the intestine and kidneys (Broer et al., 2011; Javed and Broer, 2019).

The effects of *SLC6A19* mutations on pregnancy have not been reported in humans or mice. Here, we show in mice that *Slc6a19* loss-of-function mutations cause NAD deficiency and adverse pregnancy outcomes via gene–environment interactions, as seen with enzymatic NAD synthesis genes. This is the first time a non-enzymatic component of NAD metabolism has been shown to contribute to CNDD, which is characterised by multiple malformations and embryo loss. Our findings expand our understanding of the causes behind insufficient NAD levels during embryonic development, and demonstrate that genes involved in the absorption, transport and processing of precursors or co-factors required for NAD synthesis may be candidates for CNDD in humans.

### Adverse pregnancy outcomes result from maternal, not embryonic, *Slc6a19* deficiency

Consistent with previous work (Broer et al., 2011), heterozygous-null *Slc6a19* mice had no adverse phenotype when pregnant females are maintained on the standard diet (Table 1). Accordingly, their circulatory tryptophan and all other NAD-related precursor levels were largely unaffected at both E9.5 and E11.5 (Fig. 3). This indicates that the effect of maternal *Slc6a19* heterozygosity on tryptophan provision to embryos can be overcome when excess dietary tryptophan and vitamin B3 are available, because renal tryptophan reabsorption likely remains complete on a diet replete in tryptophan and reduced intestinal absorption might be compensated for by the length of the absorptive surface in the intestine. However, we found that total NAD concentrations were significantly reduced in the whole blood of *Slc6a19*^+/−^ mothers on standard diet compared to wild-type mothers on the same diet (Fig. 2D). This might be an innate adaptation to *Slc6a19* heterozygosity and requires further investigation.

Yet, when limited tryptophan is provided in the absence of other NAD sources, as in our restricted diets, maternal *Slc6a19* heterozygosity exacerbates NAD deficiency and increases embryo death relative to wild-type mothers under the same conditions (Table 1), like the gene–environment interaction seen with *Haao*^+/−^ mice (Cuny et al., 2020). But, in contrast to *Haao*^+/−^ mice, *Slc6a19* embryonic genotype had no bearing on the survival, malformation incidence, weight or NAD status of embryos. Therefore, embryos do not appear to require *Slc6a19* to absorb tryptophan from the maternal circulation. Instead, they use a combination of active transporters of the Slc38 family on the maternal side of the placenta and passive transporters on the foetal side, in combination with amino acid exchangers (4F2hc, LAT1 and LAT2; encoded by *Slc3a2*, *Slc7a5* and *Slc7a8*, respectively), to achieve vectorial transport (Broer, 2014; Gaccioli et al., 2015; Grillo et al., 2008). The adverse pregnancy outcomes are therefore primarily caused by the reduced capacity of *Slc6a19*^+/−^ mothers to absorb and retain tryptophan via the intestines and kidneys when dietary tryptophan is limited, resulting in insufficient provision of tryptophan and other NAD precursors to the embryos. It is worth noting that although the peptide transporters PEPT1 (encoded by *Slc15a1*) and PEPT2 (encoded by *Slc15a2*) normally contribute to tryptophan absorption (Nassl et al., 2011), and likely can compensate for amino acid depletion in Hartnup disorder (Broer, 2009; Daniel, 2004), our restricted diet only provides tryptophan as amino acid and not as protein.

### The NAD metabolome of pregnant *Slc6a19*^+/−^ and wild-type mice differs in response to tryptophan restriction

NAD *de novo* synthesis from tryptophan is predominantly performed in the liver, with a small contribution by the kidneys (Liu et al., 2018). These organs export the Salvage pathway metabolite nicotinamide into the circulation, where it is used to maintain normal NAD levels in other tissues (Liu et al., 2018). Under dietary NAD precursor restriction (low tryptophan, negligible vitamin B3), total liver NAD and circulating nicotinamide were reduced equally at all investigated gestational stages, regardless of genotype (Figs 2 and 3). This suggests that the worse pregnancy outcomes in *Slc6a19^+/^*^−^ mothers do not arise from reduced *de novo* NAD synthesis activity in the liver or the nicotinamide output of the liver. Although embryos from *Slc6a19*^+/−^ mothers are more likely to die *in utero* relative to those from wild-type mothers when provided with the restricted diet, both are heterogeneous in phenotype from litter to litter (Figs S2, S5 and S6). Maternal tryptophan, kynurenine, NAD^+^ and NMN levels differed based on litter phenotype, whereby entire litter resorptions coincided with a decline in multiple NAD metabolites, confirming that they were under the greatest metabolic stress (Figs S8, S13 and S14).

### NAD metabolome perturbations have different effects at different developmental timepoints

Human patients and mice with biallelic loss of function of *HAAO*, *KYNU* or *NADSYN1* develop CNDD. However, with sufficient vitamin B3 vitamers, *Haao*-null mouse embryos can develop normally, suggesting that NAD *de novo* synthesis occurs in an embryonic-derived tissue and that embryos do not need to perform NAD *de novo* synthesis if other dietary NAD precursors downstream of the genetic block are available (Cuny et al., 2020; Shi et al., 2017). This also suggests that changes to the maternal NAD precursor supply to the embryo can have different impacts depending on when specific NAD synthesis pathways become active in the embryo. The restricted diet caused a significant drop in maternal circulatory tryptophan concentrations in both wild-type and *Slc6a19*^+/−^ mothers, but at different gestational stages, E9.5 and E11.5, respectively (Table 4, Fig. 3). Although, there was no difference in total embryo NAD levels between wild-type and *Slc6a19*^+/−^ litters on the restricted diet at E9.5, NAD levels in embryos from *Slc6a19*^+/−^ litters were slightly lower at E11.5 (Table 2, Fig. 2A,B). This suggests that reduced circulating tryptophan impairs embryonic NAD *de novo* synthesis at E11.5, but not at E9.5, because the pathway is activated in embryos between these stages.

We also observed that reduced maternal circulatory tryptophan concentration correlated with decreased embryo size for gestational age. In wild-type mothers on the restricted diet, tryptophan levels at E9.5 were significantly lower than those in *Slc6a19^+/−^* mothers, and developmental delay was primarily observed in embryos from wild-type mothers at E9.5. However, tryptophan levels in wild-type mothers returned to normal by E11.5, and embryos appeared normally sized at E18.5. In contrast, *Slc6a19^+/−^* mothers on the restricted diet had significantly lower tryptophan levels at E11.5 (Fig. 3, Table 4). This suggests that *Slc6a19* heterozygosity affects the maternal capacity to retain enough circulating tryptophan for normal embryonic growth in mid-gestation. In addition, a significant proportion of embryos were small for their gestation age at E18.5 (Fig. 1B; Table S3). It is possible that restriction of maternal circulatory tryptophan provision is causative for the small embryo size at late gestation, when tryptophan demand for embryonic development increases (Xu et al., 2017). Reduced embryo size was more prevalent among embryos from *Slc6a19*^+/−^ mothers on the restricted diet than among those from *Slc6a19*^+/−^ mothers on the standard diet (Fig. 1B), which might suggest that tryptophan limitation was the cause of the growth limitation. Additionally, placental defects have been linked to abnormal embryo development (Perez-Garcia et al., 2018; Woods et al., 2018), and it is possible that placental anomalies contributed to the observed phenotype. Preliminary assessments of placental structure and vascularisation did not reveal morphological differences between placentas from affected and normal E18.5 embryos (Fig. S4). Further work is required to better understand the cause of the observed embryonic growth restriction.

### Timing of embryonic NAD deficiency causes malformations in different organs

**Fig. 4.**
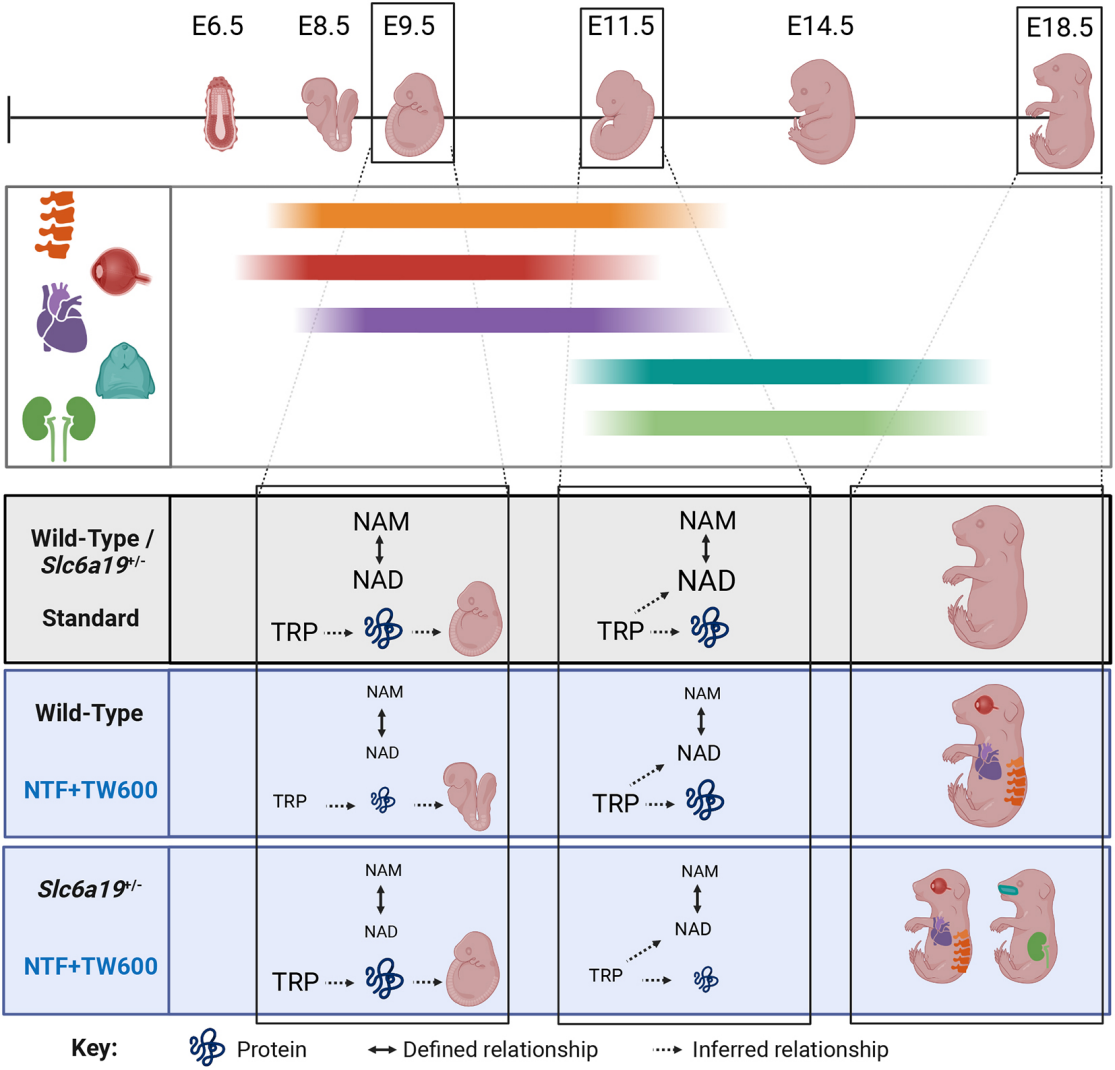
**A model to explain how congenital malformations and developmental delay arise from distinct mechanisms when dietary NAD precursors are restricted to the developing embryo.** Organogenesis is a staggered process, with different organs developing at different times in gestation. The timing of embryonic NAD deficiency under NTF+TW600 (tryptophan-restricted diet) influences the types of defects that arise. At E9.5, maternal circulatory nicotinamide is the primary source of NAD provided to the embryos and is similarly low in both maternal genotypes. This results in embryo NAD levels being equally reduced at developmental timepoints critical to eye, heart and vertebral formation, resulting in their coincidental malformation at similar rates between offspring of wild-type and *Slc6a19*^+/−^ mothers. At E11.5, circulating nicotinamide levels remain low in both maternal genotypes, but circulating tryptophan levels are specifically reduced in *Slc6a19*^+/−^ mothers, likely due to inefficient absorption from the diet combined with increasing demand for protein synthesis as embryos grow. This drop in circulatory tryptophan coincides with a minor reduction in embryo NAD levels in *Slc6a19^+/−^* litters relative to those in wild-type litters. It suggests that NAD *de novo* synthesis is active in the embryo at E11.5 and that embryo NAD levels at this stage are determined by both maternal nicotinamide and tryptophan availability. Reduced E11.5 embryonic NAD levels are assumed to cause defects in organs that develop later during organogenesis, such as kidneys and palate. This is presumably independent of the early NAD deficiency and only affects a subset of *Slc6a19*^+/−^ litters (see Fig. 1D). In addition, it is assumed that at all stages of development, tryptophan availability has direct effects on protein synthesis, with the differential timing of reduced tryptophan in wild-type and *Slc6a19*^+/−^ mothers causing developmental delay at E9.5 and E18.5, respectively. Figure created with BioRender.com. NAM, nicotinamide; TRP, tryptophan.

Organs and structures have individual developmental trajectories with distinct and coincident timing during gestation (Fig. 4). Therefore, the timing of NAD deficiency is likely to cause defects in organs/structures that are formed coincidentally. Heart, eye and vertebral defects frequently co-occurred in both *Slc6a19*^+/−^ and wild-type litters fed the restricted diet (Fig. 1C,D), and these defect types occurred in similar incidences in both maternal genotypes. These organs all develop during early organogenesis (Fig. 4), suggesting that early NAD deficiency drives the formation of these malformations. In agreement with this, embryo NAD levels were similarly perturbed in both wild-type and *Slc6a19*^+/−^ litters at E9.5, during critical stages of their formation. These ‘early’ malformations were mostly absent in an additional subset of malformed embryos from *Slc6a19*^+/−^ litters, whereas kidney hypoplasia and cleft palate were prevalent (Fig. 1D). Yet, in wild-type litters, kidney hypoplasia affected only 1/92 embryos, and cleft palate was not observed at all (Fig. 1C). Palate and kidneys develop at later stages of organogenesis (Fig. 4), suggesting that NAD deficiency occurred later in this subset of embryos. At E11.5, average embryo NAD levels were slightly lower in *Slc6a19*^+/−^ litters than in wild-type litters. This suggests that the subset of embryos from *Slc6a19*^+/−^ mothers exhibiting primarily ‘late’ malformations (kidney hypoplasia, cleft palate) might have experienced more severe NAD deficiency at a later stage compared to those exhibiting the ‘earlier’ malformation types. However, because embryos could only be dissected either at mid-gestation for NAD measurement or at E18.5 for phenotyping, it can only be speculated what the NAD status of a phenotyped E18.5 embryo had been at earlier stages of development.

The variability in severity of embryo outcomes among both *Slc6a19*^+/−^ and wild-type litters likely results from inherent metabolic differences between females and their ability to adapt to the dietary nutrient restriction. This inherent litter-to-litter variability prevents us from definitively linking timing of NAD deficiency at a given gestation timepoint to specific phenotypic outcomes. Further work is required to induce NAD deficiency at specific gestational stages and/or monitor the development of individual embryos over time, to confirm our observations and better understand this heterogeneity in phenotype.

### Non-enzymatic components of NAD synthesis can affect the NAD metabolome and contribute to CNDD – clinical relevance

Damaging mutations in genes of the NAD synthesis pathways that result in perturbation of the NAD metabolome are a cause of congenital malformations and recurrent miscarriage, and such malformations are collectively called CNDD (Shi et al., 2017; Szot et al., 2020, 2021). Although *Slc6a19* is not a core NAD synthesis pathway gene, our data show that *Slc6a19* heterozygosity in combination with a diet restricted in NAD precursors can perturb the NAD metabolome and cause CNDD in mice. Furthermore, altered circulatory tryptophan levels during pregnancy have additional implications on embryo survival and development.

Approximately one in 578 people carry a predicted loss-of-function variant in *SLC6A19*, according to the Genome Aggregation Database (gnomAD), comprised of data from 141,456 individuals without severe paediatric diseases (Karczewski et al., 2020). Female carriers of such *SLC6A19* mutations might be predisposed to an adverse pregnancy outcome if there is insufficient tryptophan or vitamin B3 available to generate NAD during gestation. Therefore, the findings of this study expand our understanding of the complex metabolic processes during pregnancy and the causation of congenital malformations. They support the notion that any gene involved in the absorption, transport and/or processing of precursors or co-factors required for NAD synthesis should be considered as a potential contributor to CNDD.

## MATERIALS AND METHODS

### Animal experiments

All animal experiments were performed in accordance with protocols approved by the Garvan Institute of Medical Research/St Vincent's Animal Experimentation Ethics Committee, Sydney, Australia (approvals 15/27 and 18/27). Mice with *Slc6a19* loss-of-function mutation on a C57BL/6J background have been described previously (Broer et al., 2011).

Mice were treated with special diets and timed mated as described previously (Cuny et al., 2020). Briefly, female mice were provided standard diet, a feed with defined composition (AIN93G; Specialty Feeds, Glen Forrest, Australia), for at least 3 weeks prior to dissection. From the start of pregnancy, the food was replaced with a feed restricted in NAD precursors vitamin B3 and tryptophan [NAD precursor vitamin-depleted and tryptophan-free feed (NTF); SF16-097; Specialty Feeds] and drinking water that contained defined concentrations of tryptophan [tryptophan-supplemented water (TW)], as described previously (Cuny et al., 2020) and summarised in Table S1.

Pregnant mice were culled to collect the deciduae at E7.5 and embryos at later stages up to E18.5. The number of dead embryos in the litters was recorded. E7.5 deciduae and embryos collected at E9.5 and E11.5 were used for NAD quantification, and E18.5 embryos were used for dissection and phenotyping (see below). Maternal liver, whole blood and plasma were collected at the time of embryo collection and used for analysis via enzymatic NAD assays (see below) and UHPLC-MS/MS as described previously (Cuny et al., 2021).

To obtain data for the reference embryo weight at different embryonic stages, pregnant wild-type C57BL/6J females were maintained on a mouse-breeding diet containing ∼90 mg/kg nicotinic acid, 3.7 g/kg tryptophan, totalling ∼592 μg of NAD precursors/day (Rat and Mouse Premium Breeder Diet, Gordons Specialty Feeds, Bargo, Australia), dissected at E16.5-E18.5 and their embryos weighed.

### Genotyping

Genotyping was performed using polymerase chain reaction (PCR) amplification of DNA extracted from ear clips (adult mice), lung tissue (E18.5 embryos) or yolk sacs (E9.5 and E11.5 embryos) The genotyping forward primers were 5′-CCAGTACGCCTTGCACAGTGCCATC-3′ for the wild-type allele and 5′-GGGTGGGATTAGATAAATGCCTGCTCT-3′ for the *Slc6a19* mutant allele. The reverse primer for the wild-type and *Slc6a19* mutant alleles was 5′-TTCCCTTGTGTAGGGCAAGTGGCTC-3′. PCR products were 201 and 485 base pairs in length for the wild-type and mutant alleles, respectively. E18.5 embryos were sexed by using *Sry* primers 5′-TTCAGCCCTACAGCCACATGA-3′ (forward) and 5′-ATGTGGGTTCCTGTCCCACTG-3′ (reverse) as described previously (Rodriguez et al., 2004).

### Embryo phenotyping

E18.5 embryo phenotyping was done as described previously (Cuny et al., 2020). Briefly, pregnant mice were sacrificed at E18.5 by cervical dislocation, live and dead embryos were counted, and live embryos were weighed and sacrificed by decapitation. General embryo morphology was assessed with light microscopy, allowing to see structural malformations of the neural tube (exencephaly), eyes, tail (caudal agenesis), limbs, digits, palate and abdominal wall. Hearts were dissected and assessed for structural malformations using optical projection tomography as described previously (Shi et al., 2016). Lengths of dissected kidneys were measured with the grid of a haemocytometer. Skeletal morphology was examined following an Alcian Blue/Alizarin Red double-staining protocol modified from Wallin et al. (1994), as described (Shi et al., 2017). Embryos were photographed using an M125 microscope (Leica Microsystems, Wetzlar, Germany). To quantify embryo outcomes, we grouped malformation types according to the body part or organ they affected, counted the number of affected organs, and classified embryos that had one or more malformations as malformed.

### NAD quantification by enzymatic cycling assay

Total NAD levels (NAD^+^ and NADH) of E9.5 and E11.5 whole embryos, E7.5 whole deciduae, and maternal liver collected at all three timepoints (E7.5, E9.5, E11.5) were measured with an enzymatic cycling assay as described previously (Cuny et al., 2020). The same assay with minor adjustments was used to measure the NAD concentration in whole-blood samples. Briefly, 30 µl whole blood was diluted in 570 µl 0.2 M NaOH+1% dodecyltrimethylammonium bromide and vortex mixed, and 100 µl of the mixture was diluted in an equal volume of a 0.4 M HCl and 0.5 M Tris base solution, vortex mixed again and centrifuged for 10 min at 15,000 ***g*** at 4°C to remove debris. The supernatant was transferred into a new tube, and 10 µl of it was measured with the enzymatic cycling assay, as done for embryos and tissue samples, as described previously (Cuny et al., 2020).

### Metabolite quantification by UHPLC-MS/MS

NAD-related metabolites tryptophan, kynurenine, nicotinamide, NMN, NAD^+^, 2PY and 4PY were quantified in plasma and whole blood of pregnant mice collected at E9.5 and E11.5 using UHPLC-MS/MS as described previously (Cuny et al., 2021).

### Statistical analysis

Two-sided Fisher's exact test was used to compare rates of defects and embryo loss between two treatment groups of mice, with Freeman-Halton extension (2×3 contingency table) when three groups were compared. Chi-squared test was used for comparing multiple groups. Total NAD levels, NAD-related metabolite levels and embryo weights were analysed by one-way ANOVA with Tukey's multiple comparisons test, and statistical significance relative to the wild-type on standard diet control group was reported. The effects of metabolite levels on litter survival were assessed by ordinary two-way ANOVA. Two-way ANOVA with Tukey's multiple comparison test was used to assess the effects of embryo genotype on total NAD levels within each diet group. Three-way ANOVA was performed across the two gene–environment conditions (wild type and *Slc6a19*^+/−^) and three timepoints (E7.5, E9.5, E11.5) to compare the effect of maternal genotype, diet and stage of gestation on maternal NAD levels globally. Embryo weights, NAD levels and NAD-related metabolite levels are displayed as mean±s.d. *P*<0.05 was considered significant. All statistical analyses were performed with Prism (version 8; GraphPad Software) except for Fisher's exact test with Freeman-Halton extension, for which an online tool was used (https://www.danielsoper.com/statcalc/).

## Supplementary Material

10.1242/dmm.049647_sup1 Supplementary information

## References

[DMM049647C1] Baron, D. N., Dent, C. E., Harris, H., Hart, E. W. and Jepson, J. B. (1956). Hereditary pellagra-like skin rash with temporary cerebellar ataxia, constant renal amino-aciduria, and other bizarre biochemical features. *Lancet* 271, 421-428. 10.1016/S0140-6736(56)91914-6 13358233

[DMM049647C2] Broer, A., Klingel, K., Kowalczuk, S., Rasko, J. E., Cavanaugh, J. and Broer, S. (2004). Molecular cloning of mouse amino acid transport system B0, a neutral amino acid transporter related to Hartnup disorder. *J. Biol. Chem.* 279, 24467-24476. 10.1074/jbc.M400904200 15044460

[DMM049647C3] Broer, A., Juelich, T., Vanslambrouck, J. M., Tietze, N., Solomon, P. S., Holst, J., Bailey, C. G., Rasko, J. E. and Broer, S. (2011). Impaired nutrient signaling and body weight control in a Na+ neutral amino acid cotransporter (Slc6a19)-deficient mouse. *J. Biol. Chem.* 286, 26638-26651. 10.1074/jbc.M111.241323 21636576 PMC3143628

[DMM049647C4] Broer, S. (2009). The role of the neutral amino acid transporter B0AT1 (SLC6A19) in Hartnup disorder and protein nutrition. *IUBMB Life* 61, 591-599. 10.1002/iub.210 19472175 PMC7165679

[DMM049647C5] Broer, S. (2014). The SLC38 family of sodium-amino acid co-transporters. *Pflugers Arch.* 466, 155-172. 10.1007/s00424-013-1393-y 24193407

[DMM049647C6] Broer, S., Cavanaugh, J. A. and Rasko, J. E. (2005). Neutral amino acid transport in epithelial cells and its malfunction in Hartnup disorder. *Biochem. Soc. Trans.* 33, 233-236. 10.1042/BST0330233 15667315

[DMM049647C7] Cuny, H., Rapadas, M., Gereis, J., Martin, E., Kirk, R. B., Shi, H. and Dunwoodie, S. L. (2020). NAD deficiency due to environmental factors or gene-environment interactions causes congenital malformations and miscarriage in mice. *Proc. Natl. Acad. Sci. USA* 117, 3738-3747. 10.1073/pnas.1916588117 32015132 PMC7035598

[DMM049647C8] Cuny, H., Kristianto, E., Hodson, M. P. and Dunwoodie, S. L. (2021). Simultaneous quantification of 26 NAD-related metabolites in plasma, blood, and liver tissue using UHPLC-MS/MS. *Anal. Biochem.* 633, 114409. 10.1016/j.ab.2021.114409 34648806

[DMM049647C9] Daniel, H. (2004). Molecular and integrative physiology of intestinal peptide transport. *Annu. Rev. Physiol.* 66, 361-384. 10.1146/annurev.physiol.66.032102.144149 14977407

[DMM049647C10] Dolle, C., Skoge, R. H., Vanlinden, M. R. and Ziegler, M. (2013). NAD biosynthesis in humans--enzymes, metabolites and therapeutic aspects. *Curr. Top. Med. Chem.* 13, 2907-2917. 10.2174/15680266113136660206 24171775

[DMM049647C11] Ford, H. B. and Schust, D. J. (2009). Recurrent pregnancy loss: etiology, diagnosis, and therapy. *Rev. Obstet. Gynecol.* 2, 76-83. 19609401 PMC2709325

[DMM049647C12] Gaccioli, F., Aye, I. L., Roos, S., Lager, S., Ramirez, V. I., Kanai, Y., Powell, T. L. and Jansson, T. (2015). Expression and functional characterisation of System L amino acid transporters in the human term placenta. *Reprod. Biol. Endocrinol.* 13, 57. 10.1186/s12958-015-0054-8 26050671 PMC4462079

[DMM049647C13] Griffiths, H. B. S., Williams, C., King, S. J. and Allison, S. J. (2020). Nicotinamide adenine dinucleotide (NAD+): essential redox metabolite, co-substrate and an anti-cancer and anti-ageing therapeutic target. *Biochem. Soc. Trans.* 48, 733-744. 10.1042/BST20190033 32573651

[DMM049647C14] Grillo, M. A., Lanza, A. and Colombatto, S. (2008). Transport of amino acids through the placenta and their role. *Amino Acids* 34, 517-523. 10.1007/s00726-007-0006-5 18172742

[DMM049647C15] Hobbs, C. A., Chowdhury, S., Cleves, M. A., Erickson, S., MacLeod, S. L., Shaw, G. M., Shete, S., Witte, J. S. and Tycko, B. (2014). Genetic epidemiology and nonsyndromic structural birth defects: from candidate genes to epigenetics. *JAMA Pediatr*. 168, 371-377. 10.1001/jamapediatrics.2013.4858 24515445 PMC3981910

[DMM049647C16] Houtkooper, R. H., Canto, C., Wanders, R. J. and Auwerx, J. (2010). The secret life of NAD+: an old metabolite controlling new metabolic signaling pathways. *Endocr. Rev.* 31, 194-223. 10.1210/er.2009-0026 20007326 PMC2852209

[DMM049647C17] Javed, K. and Broer, S. (2019). Mice lacking the intestinal and renal neutral amino acid transporter SLC6A19 demonstrate the relationship between dietary protein intake and amino acid malabsorption. *Nutrients* 11, 2024. 10.3390/nu11092024 31470570 PMC6770948

[DMM049647C18] Karczewski, K. J., Francioli, L. C., Tiao, G., Cummings, B. B., Alfoldi, J., Wang, Q., Collins, R. L., Laricchia, K. M., Ganna, A., Birnbaum, D. P. et al. (2020). The mutational constraint spectrum quantified from variation in 141,456 humans. *Nature* 581, 434-443. 10.1038/s41586-020-2308-7 32461654 PMC7334197

[DMM049647C19] Khoury, M. J. and Erickson, J. D. (1993). Recurrent pregnancy loss as an indicator for increased risk of birth defects: a population-based case-control study. *Paediatr. Perinat. Epidemiol.* 7, 404-416. 10.1111/j.1365-3016.1993.tb00422.x 8290380

[DMM049647C20] Kleta, R., Romeo, E., Ristic, Z., Ohura, T., Stuart, C., Arcos-Burgos, M., Dave, M. H., Wagner, C. A., Camargo, S. R., Inoue, S. et al. (2004). Mutations in SLC6A19, encoding B0AT1, cause Hartnup disorder. *Nat. Genet.* 36, 999-1002. 10.1038/ng1405 15286787

[DMM049647C21] Liu, L., Su, X., Quinn, W. J., III, Hui, S., Krukenberg, K., Frederick, D. W., Redpath, P., Zhan, L., Chellappa, K., White, E. et al. (2018). Quantitative analysis of NAD synthesis-breakdown fluxes. *Cell Metab.* 27, 1067-1080.e5. 10.1016/j.cmet.2018.03.018 29685734 PMC5932087

[DMM049647C22] Moorthie, S., Blencowe, H., Darlison, M. W., Lawn, J., Morris, J. K., Modell, B., Congenital Disorders Expert Group, Bittles, A. H., Blencowe, H., Christianson, A. et al. (2018). Estimating the birth prevalence and pregnancy outcomes of congenital malformations worldwide. *J. Community Genet* 9, 387-396. 10.1007/s12687-018-0384-2 30218347 PMC6167261

[DMM049647C23] Nassl, A. M., Rubio-Aliaga, I., Fenselau, H., Marth, M. K., Kottra, G. and Daniel, H. (2011). Amino acid absorption and homeostasis in mice lacking the intestinal peptide transporter PEPT1. *Am. J. Physiol. Gastrointest. Liver Physiol.* 301, G128-G137. 10.1152/ajpgi.00017.2011 21350187

[DMM049647C24] Palou, A., Arola, L. and Alemany, M. (1977). Plasma amino acid concentrations in pregnant rats and in 21-day foetuses. *Biochem. J.* 166, 49-55. 10.1042/bj1660049 901417 PMC1164955

[DMM049647C25] Perez-Garcia, V., Fineberg, E., Wilson, R., Murray, A., Mazzeo, C. I., Tudor, C., Sienerth, A., White, J. K., Tuck, E., Ryder, E. J. et al. (2018). Placentation defects are highly prevalent in embryonic lethal mouse mutants. *Nature* 555, 463-468. 10.1038/nature26002 29539633 PMC5866719

[DMM049647C26] Philipp, T., Philipp, K., Reiner, A., Beer, F. and Kalousek, D. K. (2003). Embryoscopic and cytogenetic analysis of 233 missed abortions: factors involved in the pathogenesis of developmental defects of early failed pregnancies. *Hum. Reprod.* 18, 1724-1732. 10.1093/humrep/deg309 12871891

[DMM049647C27] Rai, R. and Regan, L. (2006). Recurrent miscarriage. *Lancet* 368, 601-611. 10.1016/S0140-6736(06)69204-0 16905025

[DMM049647C28] Reeves, P. G., Nielsen, F. H. and Fahey, G. C., Jr. (1993). AIN-93 purified diets for laboratory rodents: final report of the American Institute of Nutrition ad hoc writing committee on the reformulation of the AIN-76A rodent diet. *J. Nutr.* 123, 1939-1951. 10.1093/jn/123.11.1939 8229312

[DMM049647C29] Rodriguez, T. A., Sparrow, D. B., Scott, A. N., Withington, S. L., Preis, J. I., Michalicek, J., Clements, M., Tsang, T. E., Shioda, T., Beddington, R. S. et al. (2004). Cited1 is required in trophoblasts for placental development and for embryo growth and survival. *Mol. Cell. Biol.* 24, 228-244. 10.1128/MCB.24.1.228-244.2004 14673158 PMC303371

[DMM049647C30] Sainio, E. L., Pulkki, K. and Young, S. N. (1996). L-Tryptophan: Biochemical, nutritional and pharmacological aspects. *Amino Acids* 10, 21-47. 10.1007/BF00806091 24178430

[DMM049647C31] Schröcksnadel, K., Wirleitner, B., Winkler, C. and Fuchs, D. (2006). Monitoring tryptophan metabolism in chronic immune activation. *Clin. Chim. Acta* 364, 82-90. 10.1016/j.cca.2005.06.013 16139256

[DMM049647C32] Scriver, C. R. (1965). Hartnup disease: a genetic modification of intestinal and renal transport of certain neutral alpha-amino acids. *N. Engl. J. Med.* 273, 530-532. 10.1056/NEJM196509022731005 14324515

[DMM049647C33] Seow, H. F., Broer, S., Broer, A., Bailey, C. G., Potter, S. J., Cavanaugh, J. A. and Rasko, J. E. (2004). Hartnup disorder is caused by mutations in the gene encoding the neutral amino acid transporter SLC6A19. *Nat. Genet.* 36, 1003-1007. 10.1038/ng1406 15286788

[DMM049647C34] Shi, H., Enriquez, A., Rapadas, M., Martin, E., Wang, R., Moreau, J., Lim, C. K., Szot, J. O., Ip, E., Hughes, J. N. et al. (2017). NAD deficiency, congenital malformations, and niacin supplementation. *N. Engl. J. Med.* 377, 544-552. 10.1056/NEJMoa1616361 28792876

[DMM049647C35] Shi, H., O'Reilly, V. C., Moreau, J. L., Bewes, T. R., Yam, M. X., Chapman, B. E., Grieve, S. M., Stocker, R., Graham, R. M., Chapman, G. et al. (2016). Gestational stress induces the unfolded protein response, resulting in heart defects. *Development* 143, 2561-2572. 10.1242/dev.136820 27436040

[DMM049647C36] Shibata, K. and Matsuo, H. (1989). Correlation between niacin equivalent intake and urinary excretion of its metabolites, N'-methylnicotinamide, N'-methyl-2–pyridone-5-carboxamide, and N'-methyl-4-pyridone-3-carboxamide, in humans consuming a self-selected food. *Am. J. Clin. Nutr.* 50, 114-119. 10.1093/ajcn/50.1.114 2526576

[DMM049647C37] Szot, J. O., Campagnolo, C., Cao, Y., Iyer, K. R., Cuny, H., Drysdale, T., Flores-Daboub, J. A., Bi, W., Westerfield, L., Liu, P. et al. (2020). Bi-allelic mutations in NADSYN1 cause multiple organ defects and expand the genotypic spectrum of congenital NAD deficiency disorders. *Am. J. Hum. Genet.* 106, 129-136. 10.1016/j.ajhg.2019.12.006 31883644 PMC7042491

[DMM049647C38] Szot, J. O., Slavotinek, A., Chong, K., Brandau, O., Nezarati, M., Cueto-Gonzalez, A. M., Patel, M. S., Devine, W. P., Rego, S., Acyinena, A. P. et al. (2021). New cases that expand the genotypic and phenotypic spectrum of Congenital NAD Deficiency Disorder. *Hum. Mutat.* 42, 862-876. 10.1002/humu.24211 33942433 PMC8238843

[DMM049647C39] Theiler, K. (1989). *The House Mouse: Atlas of Embryonic Development*. New York: Springer-Verlag.

[DMM049647C40] Trammell, S. A., Schmidt, M. S., Weidemann, B. J., Redpath, P., Jaksch, F., Dellinger, R. W., Li, Z., Abel, E. D., Migaud, M. E. and Brenner, C. (2016). Nicotinamide riboside is uniquely and orally bioavailable in mice and humans. *Nat. Commun.* 7, 12948. 10.1038/ncomms12948 27721479 PMC5062546

[DMM049647C41] Wallin, J., Wilting, J., Koseki, H., Fritsch, R., Christ, B. and Balling, R. (1994). The role of Pax-1 in axial skeleton development. *Development* 120, 1109-1121. 10.1242/dev.120.5.1109 8026324

[DMM049647C42] Wang, Q., Liu, D., Song, P. and Zou, M. H. (2015). Tryptophan-kynurenine pathway is dysregulated in inflammation, and immune activation. *Front. Biosci.* 20, 1116-1143. 10.2741/4363 PMC4911177 25961549

[DMM049647C43] Woods, L., Perez-Garcia, V. and Hemberger, M. (2018). Regulation of placental development and its impact on fetal growth-new insights from mouse models. *Front. Endocrinol.* 9, 570. 10.3389/fendo.2018.00570 PMC6170611 30319550

[DMM049647C44] Xu, K., Liu, H., Bai, M., Gao, J., Wu, X. and Yin, Y. (2017). Redox properties of tryptophan metabolism and the concept of tryptophan use in pregnancy. *Int. J. Mol. Sci.* 18, 1595. 10.3390/ijms18071595 28737706 PMC5536082

