# Supplementary material for: Maternal heterozygosity of Slc6a19 causes metabolic perturbation and congenital NAD deficiency disorder in mice
Source: Dis Model Mech. 2022 Nov 14;16(5):dmm049647. doi: 10.1242/dmm.049647 (PMC9702539; doi:10.1242/dmm.049647)
Supplement: Supplementary information [file dmm-16-049647-s1.pdf]

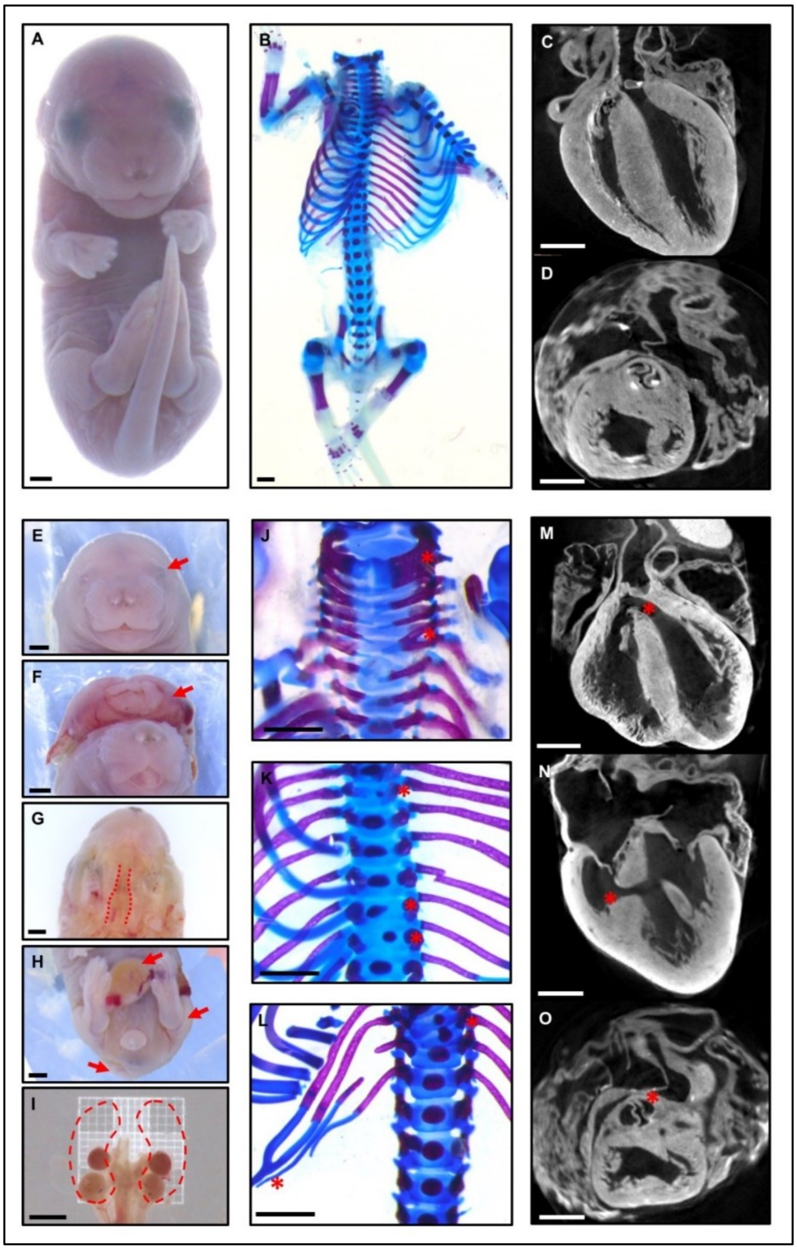

Fig. S1. Examples of malformations seen in embryos from *Slc6a19*$^{+/-}$ mothers on diets with insufficient NAD precursors. Images show E18.5 embryos. (A-D) Normal embryo, skeleton, and heart, respectively; (E) anophthalmia; (F) exencephaly; (G) cleft palate; (H) from top to bottom: intestines outside, talipes, caudal agenesis; (I) small kidneys, with comparison to normal size (dotted lines); (J-L) skeletons stained with alizarin red and alcian blue, asterisks indicate: (J) vertebral fusions, (K, top) butterfly vertebrae, (K, middle) wedge vertebrae, (K, bottom) hemivertebrae, (L, top) wedge vertebrae, (L, bottom) fused ribs; (M-O) hearts imaged with optical projection tomography, asterisks indicate: (M) membranous ventricular septal defect (VSD) with overriding aorta, (N) muscular VSD, (O) bicuspid aortic valve. Scale bar 1 mm.

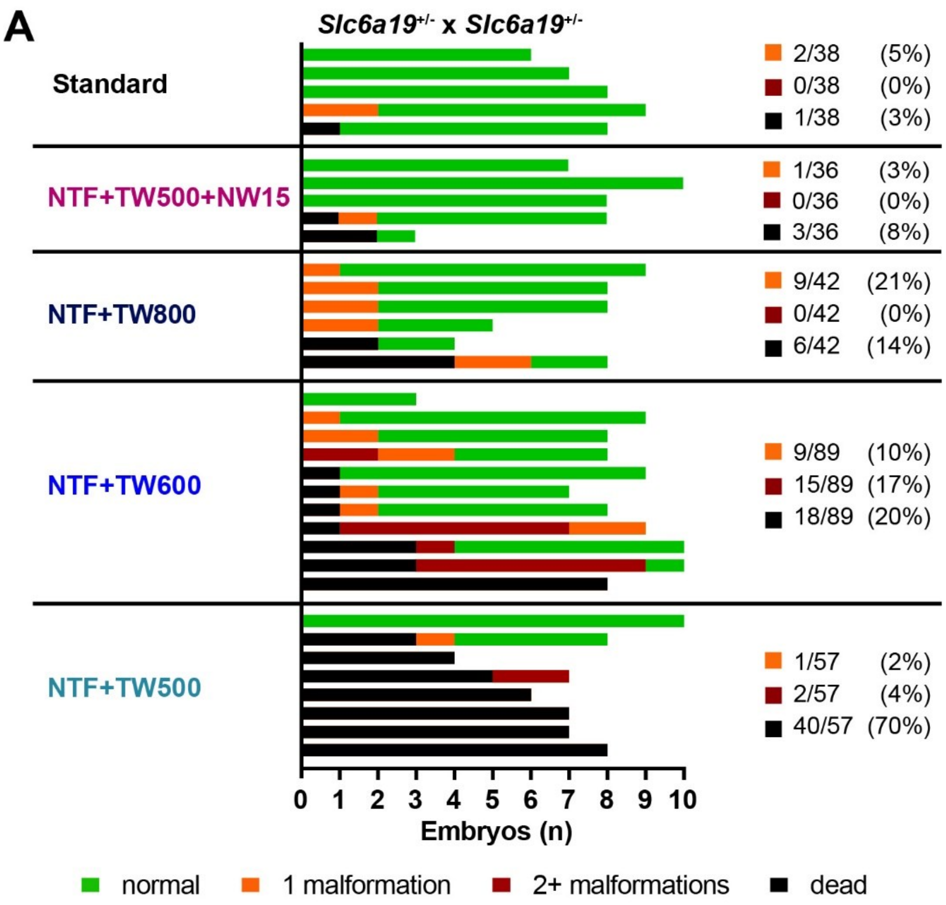

Fig. S2. Phenotypes of embryos at E18.5 from *Slc6a19*$^{+/-}$ mothers on different dietary treatments throughout pregnancy. Maternal diet conditions are indicated on the left. The mating scheme for all litters (*Slc6a19*$^{+/-}$ x *Slc6a19*$^{+/-}$) is indicated on top. Each horizontal bar represents a litter and length of the bars indicates the total number of embryos per litter. All dead embryos were found to be early resorptions. Total counts and percentages of embryos within each treatment group are summarized on the right. This dataset is related to Fig. 1A and Table 1. For the equivalent phenotype summary of litters from wild-type mothers, see Table 1 and (Cuny et al., 2020).

Disease Models & Mechanisms • Supplementary information

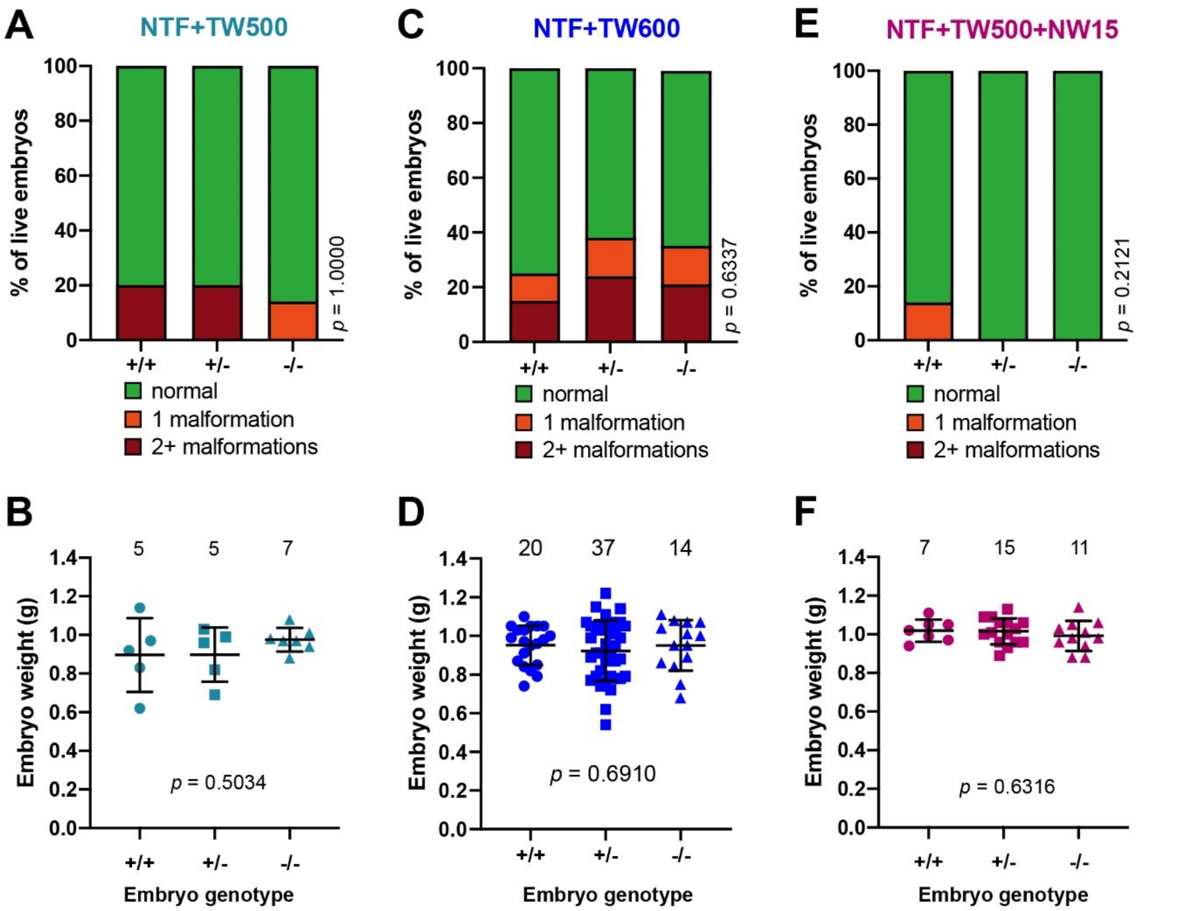

Fig. S3. Embryonic genotype in mothers with a *Slc6a19*$^{+/-}$ loss-of-function variant has no impact on embryo viability or weight. Summary of live embryo outcomes from the (**A**) NTF+TW500, (**C**) NTF +TW600 and (**E**) NTF+TW500+NW15 treatment groups sorted by embryo *Slc6a19* genotype. There is no correlation between the proportion of live embryos with malformations (orange and red) and the embryo genotype (Fisher's Exact test with Freeman-Halton extension). Dead embryos were excluded as they could not be genotyped due to complete resorption early in gestation. Comparison of embryo weights in (**B**) NTF+TW500, (**D**) NTF+TW600 and (**F**) NTF+TW500+NW15 treatment groups sorted by embryo *Slc6a19* genotype. There is no statistically significant difference in embryo weights between *Slc6a19* genotypes at E18.5 (ordinary one-way ANOVA). Bars show mean ± s.d.. Numbers of embryos per *Slc6a19* genotype are indicated above graph.

A

Genotype		Pretreatment	Pregnancy	Of all embryos		Of live embryos		
M	P	Diet	Diet	Alive	Dead	Normal	Malformed	Small
+/+	+/+	SF22-012	SF22-012	19 (79%)	5 (21%)	19 (100%)	0 (0%)	0 (0%)
+/+	+/+	SF22-012	NTF+TW600	13 (48%)	14 (52%)	11 (79%)	2 (7%)	2 (7%)
+/−	+/−	SF22-012	NTF+TW600	0 (0%)	25 (100%)	-	-	-
+/−	+/−	Breeding	NTF+TW600	16 (89%)	2 (11%)	7 (44%)	9 (56%)	1 (6%)

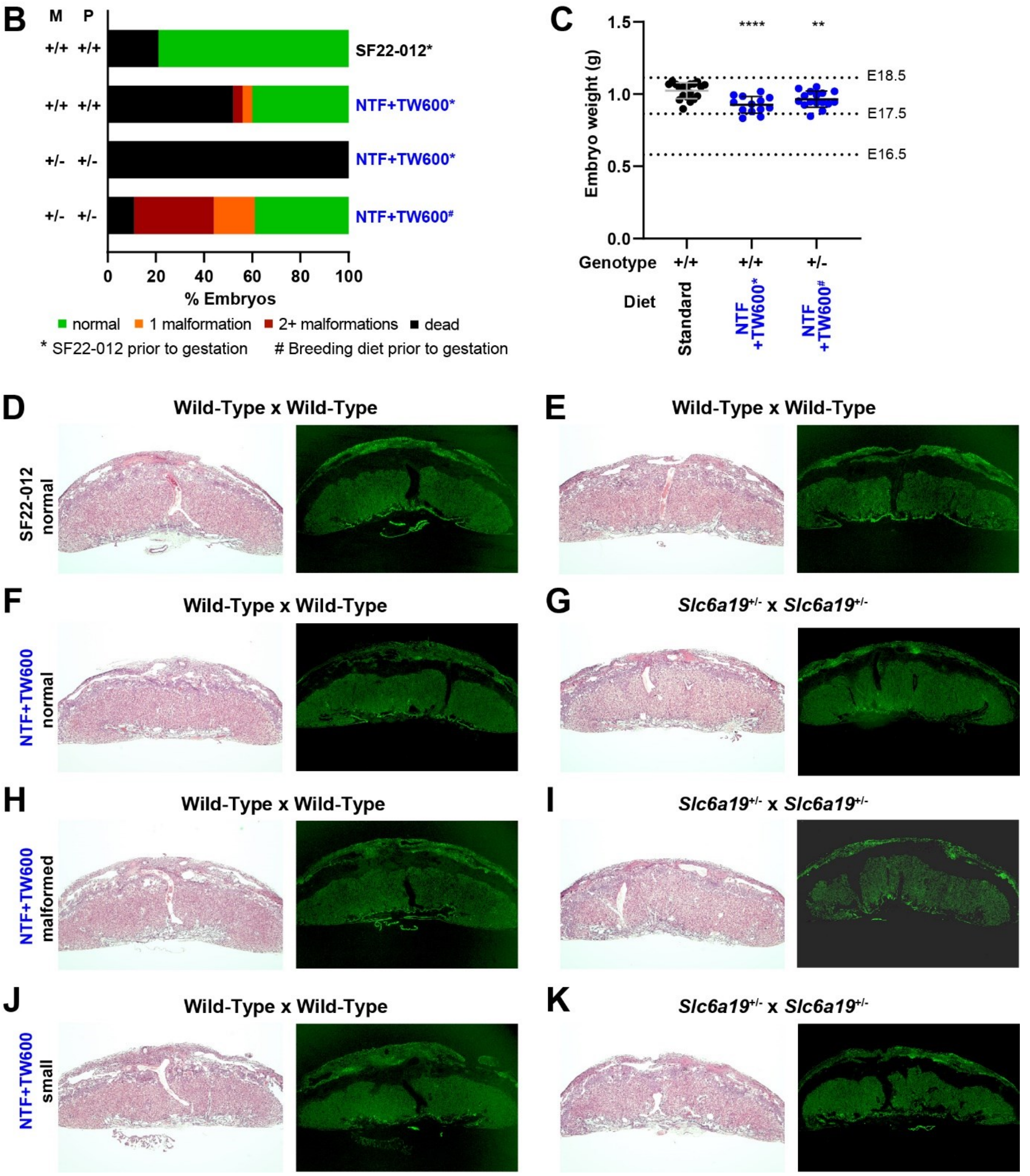

Fig. S4. The placentas of affected embryos at E18.5 have normal structure and vascularisation. (**A**) Summary of embryo phenotypic outcomes at E18.5 from wild-type x wild-type and *Slc6a19*$^{+/-}$ x *Slc6a19*$^{+/-}$ timed mating and dietary treatments for placental assessments. The maternal diet given throughout gestation was either not restricted in NAD precursors and contained 30.0 mg/kg of vitamin B3 and 1.8 g/kg of tryptophan, corresponding to 80% of the NAD precursor content of the standard diet (SF22-012; Specialty Feeds, Glen Forrest, Australia) or restricted in NAD precursors (NTF+TW600,

see Table S1). Female wild-type mice were given SF22-012 diet for at least 3 weeks prior to the start of gestation. Because embryos of *Slc6a19*$^{+/-}$ mothers pretreated with SF22-012 diet and given NTF +TW600 throughout gestation were not viable, due to their heightened sensitivity towards NAD precursor restriction, another set of *Slc6a19*$^{+/-}$ females was given the breeding diet (see Table S1) prior to gestation. NTF, NAD precursor vitamin-depleted and tryptophan-free feed; TW, tryptophan-supplemented water. M, maternal *Slc6a19* genotype; P, paternal *Slc6a19* genotype. Small embryo size was defined as weighing less that an average E17.5 embryo from a wild-type mother on the breeding diet. (**B**) Graphical representation of the embryo survival and malformation incidences. (**C**) Weights of live E18.5 embryos summarised in (**A**) and (**B**). Dissected embryos were weighed prior to assessment for congenital malformations. Bars indicate mean ± s.d.. Significance of differences relative to the wild-type SF22-012 diet control group (first column) was determined by one-way ANOVA with Tukey's multiple comparisons test with ****P<0.0001 and **P<0.01. Dotted lines indicate average weights of embryos from wild-type mothers fed the breeding diet and collected at E16.5, E17.5, and E18.5, respectively. (**D-K**) Assessment of placentas from E18.5 embryos. Multiple sections of collected placentas were processed by haematoxylin/eosin (H&E) (O'Reilly et al., 2014) and Isolectin GS-IB4 (IB4) staining. For IB4 staining, the sections were deparaffined in the same way as for H&E, and incubated 2× 5 min in PBS+0.1% TWEEN, blocked for 1 h in 1%BSA+10% horse serum at room temperature, then incubated with Isolectin GS-IB4, Alexa Fluor^{TM} 488 Conjugate (ThermoFisher Cat #I21411, 1:250 dilution) at 4°C overnight. The IB4 reagent has been validated previously (Moreau et al., 2014). Images of IB4-stained placental sections were acquired by fluorescence microscopy at 2.5× magnification using excitation/emission parameters for GFP. H&E-stained placental sections were assessed by light microscopy and images acquired at 2.5× magnification. The placentas of neither malformed embryos (**H**, **I**) nor small embryos (**J**, **K**) exhibited any obvious difference in gross structure or vascularisation beyond normal variability seen in placentas of normal embryos from wild-type x wild-type matings (**D**, **E**, **F**) or *Slc6a19*$^{+/-}$ x *Slc6a19*$^{+/-}$ matings (**G**).

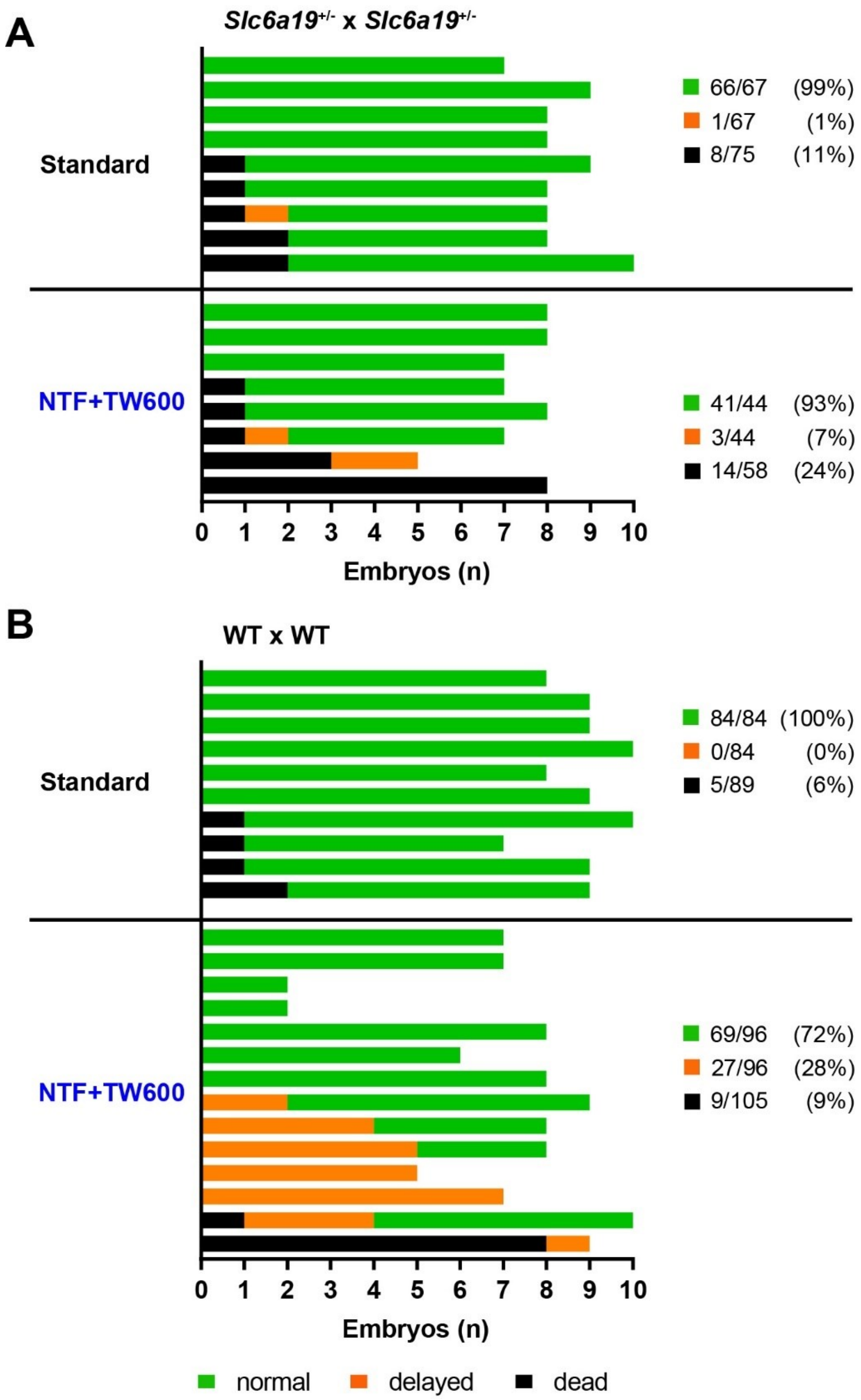

Fig. S5. Maternal dietary NAD restriction during pregnancy causes varying degrees of developmental delay that is observable at E9.5. Embryo outcomes at E9.5 with (**A**) a *Slc6a19*$^{+/-}$ x *Slc6a19*$^{+/-}$ mating scheme or (**B**) a wild-type x wild-type mating scheme and different maternal dietary conditions. Maternal diets from the start of pregnancy are indicated on the left. Each horizontal bar represents a litter and length of the bars indicates the number of embryos per litter. Total counts and percentages of embryos within each treatment group are summarized on the right. The 'delayed' group was defined as embryos phenotypically E9 or younger. WT, wild-type. This dataset is related to Table 1. Part of this dataset is from a previous study (Cuny et al., 2020).

Disease Models & Mechanisms • Supplementary information

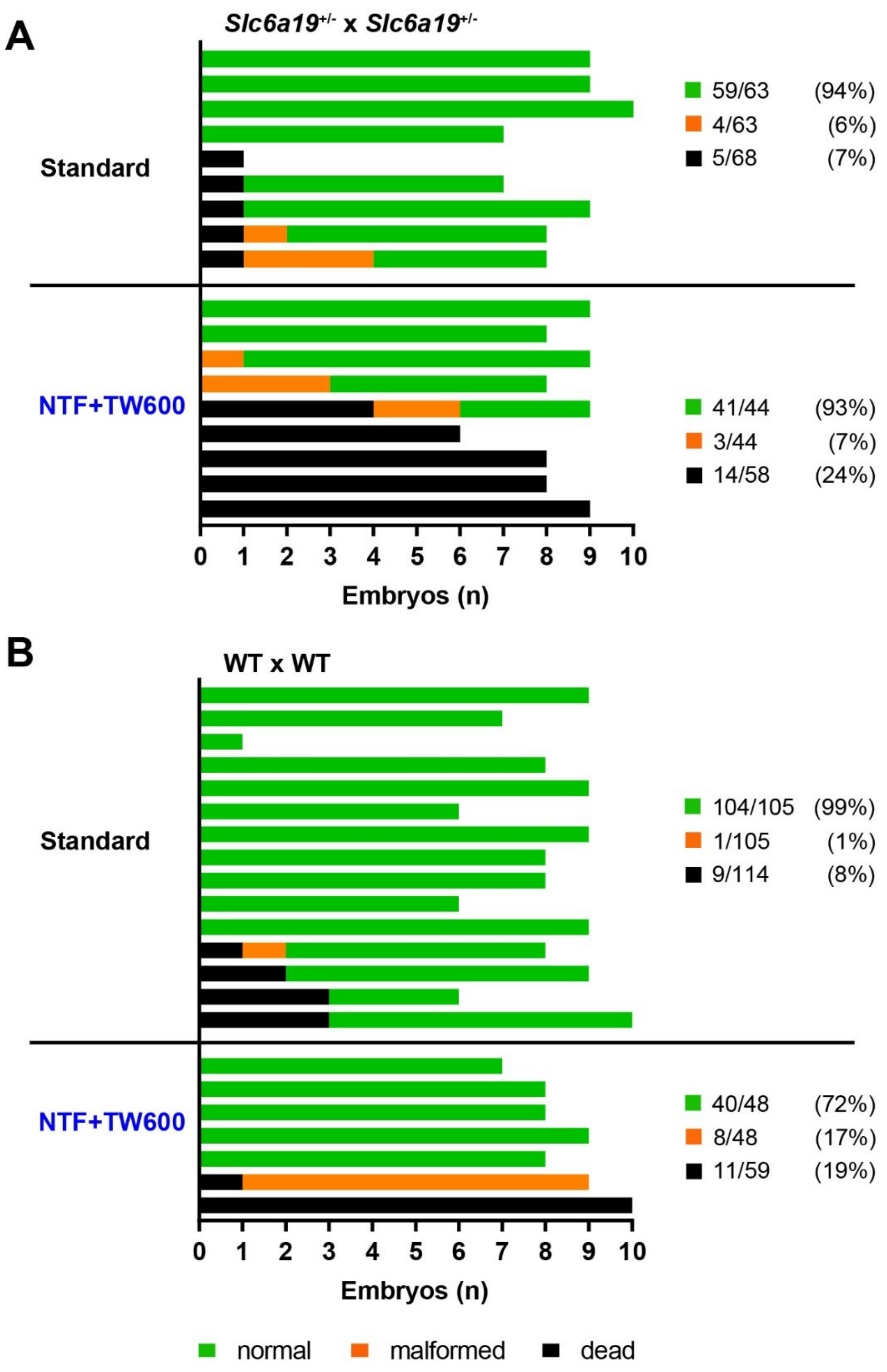

Fig. S6. Maternal dietary NAD restriction during pregnancy of *Slc6a19*$^{+/-}$ mice increases the rate of spontaneous miscarriage. Embryo outcomes at E11.5 with (**A**) a *Slc6a19*$^{+/-}$ x *Slc6a19*$^{+/-}$ mating scheme or (**B**) a wild-type x wild-type mating scheme and different maternal dietary conditions. Maternal diets from the start of pregnancy are indicated on the left. Each horizontal bar represents a litter and length of the bars indicates the number of embryos per litter. Total counts and percentages of embryos within each treatment group are summarized on the right. The 'malformed' group includes embryos with visible malformations such as short, curly, or kinked tails (caudal agenesis), and underdeveloped eyes. WT, wild-type. This dataset is related to Table 1. Part of this dataset is from a previous study (Cuny et al., 2020).

Disease Models & Mechanisms • Supplementary information

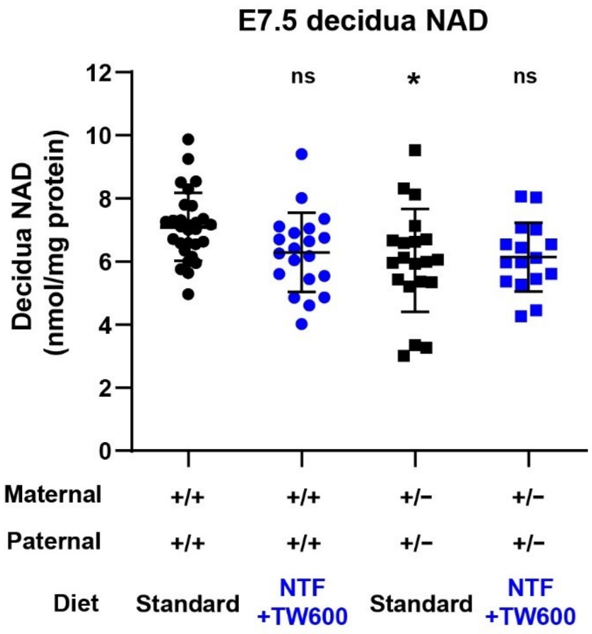

Fig. S7. Decidua total NAD (NAD^+ and NADH) concentrations at E7.5. Dots represent measured NAD concentrations, and bars indicate the mean ± s.d.. The parental *Slc6a19* genotypes and dietary conditions during pregnancy are indicated at the bottom. The deciduae were not genotyped. Significance of differences relative to the pregnant wild-type standard diet group (first column) was assessed by one-way ANOVA with Tukey's multiple comparisons test with **P*<0.05 and ns = not significant. See Table S6 for numerical values and statistics.

Disease Models & Mechanisms • Supplementary information

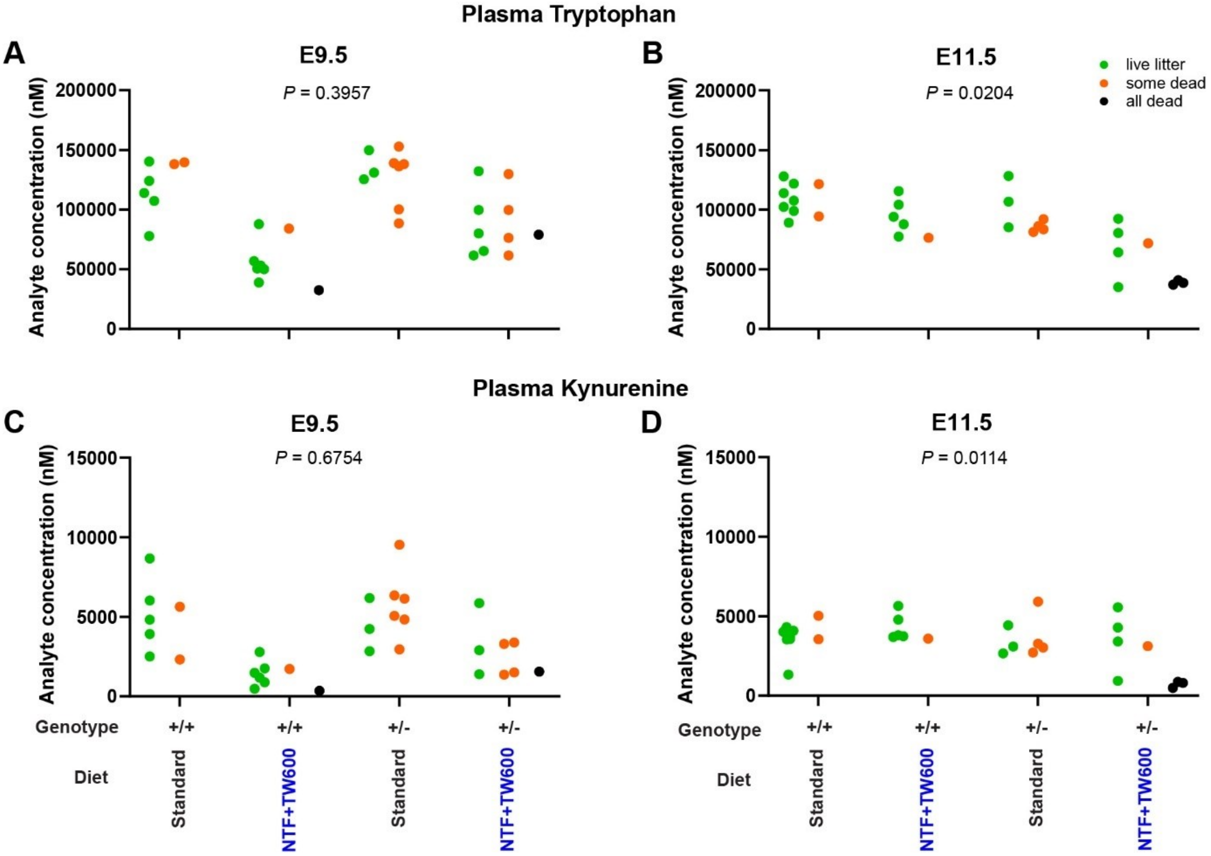

Fig. S8. Tryptophan and kynurenine concentrations in plasma of pregnant wild-type and *Slc6a19*$^{+/-}$ mice, and their correlation with the litter survival at the respective timepoints in pregnancy. Plasma metabolite concentrations at E9.5 **(A**, **C)** and at E11.5 (**B**, **D**). Colours indicate the litter survival status per mother at dissection, with green: all embryos alive, orange: one or more embryos dead, black: all embryos dead. The maternal *Slc6a19* genotype and diet are indicated underneath each graph. Trends between litter survival and maternal metabolite levels were assessed by two-way ANOVA, comparing the 3 different litter outcomes across all 4 different maternal diet/genotype conditions. *P* values are indicated within the graphs. This dataset is related to Table 4 and Fig. 3.

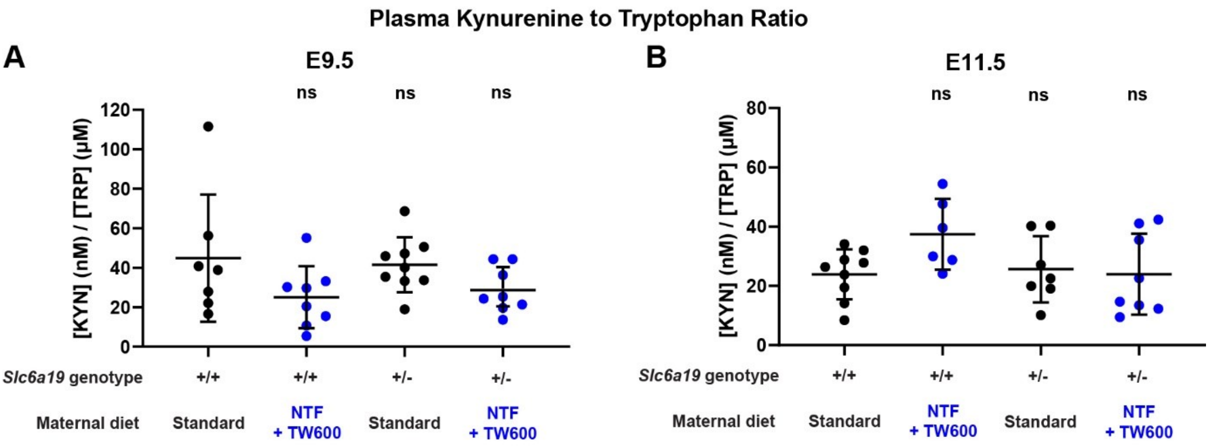

Fig. S9. Ratio of kynurenine to tryptophan concentration in plasma of pregnant wild-type and *Slc6a19*$^{+/-}$ mice. Kynurenine (nM) to tryptophan (µM) ratio (KYN:TRP) at (**A**) E9.5 and (**B**) at E11.5. Dots represent the ratio in individual mice, and bars indicate the mean ± s.d.. Significance of differences in the KYN:TRP ratio relative to the pregnant wild-type standard diet group (first column) was assessed by one-way ANOVA with Tukey's multiple comparisons test with ns = not significant. See Table 4 for numerical values and additional statistics.

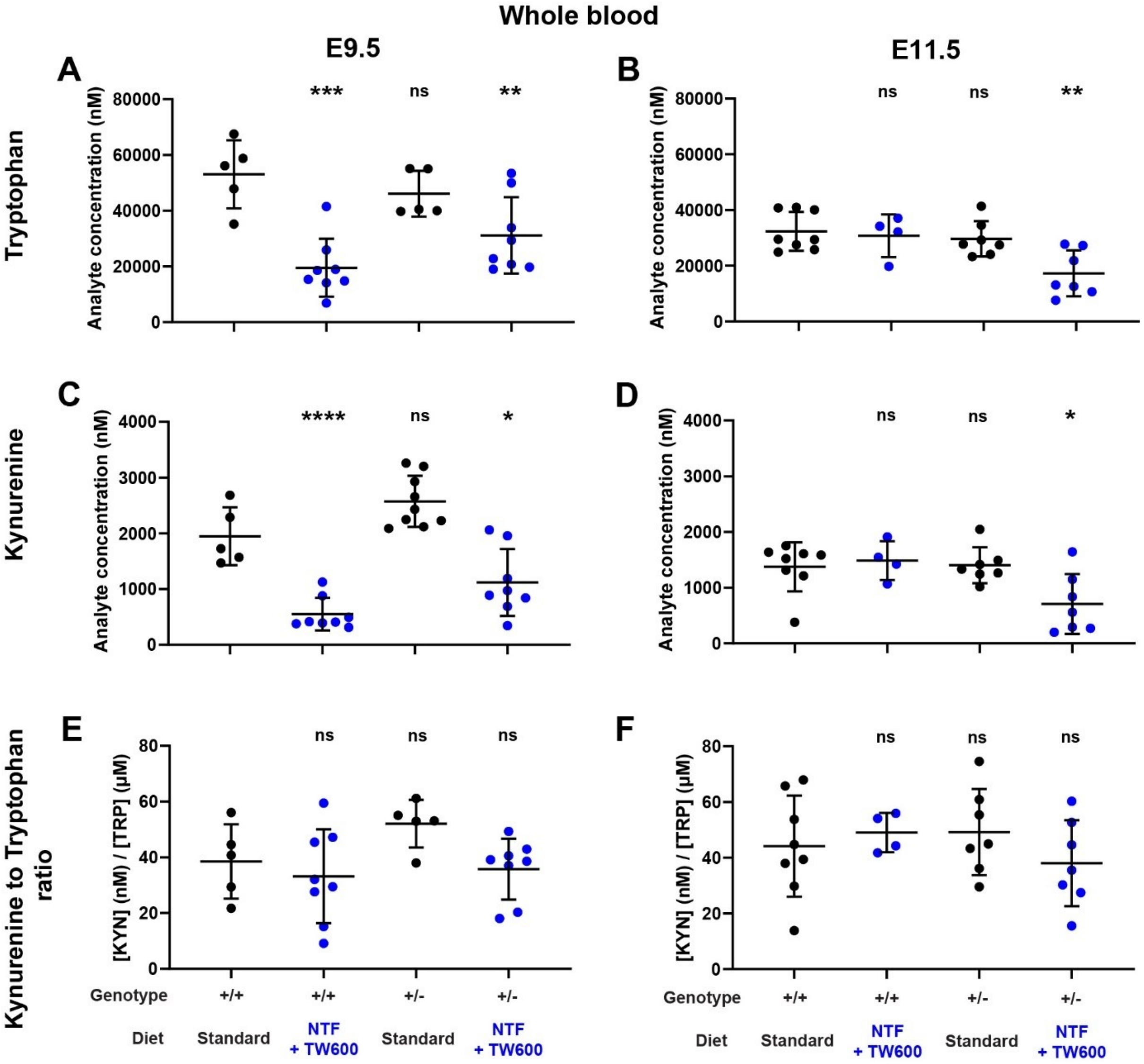

Fig. S10. Tryptophan and kynurenine concentration in whole blood of pregnant wild-type and *Slc6a19*$^{+/-}$ mice, measured by UHPLC-MS/MS. (**A**, **C**) Whole blood concentrations at E9.5; (**B**, **D**) at E11.5. Dots represent metabolite concentrations in individual mice, and bars indicate the mean ± s.d.. The maternal *Slc6a19* genotype and diet are indicated at the bottom. (**E**, **F**) Ratio of kynurenine to tryptophan (KYN:TRP) concentration in whole blood of pregnant wild-type and *Slc6a19*$^{+/-}$ mice at (**E**) E9.5 and (**F**) E11.5. Significance of differences in metabolite concentration or KYN:TRP ratio relative to the pregnant wild-type standard diet group (first column) was assessed by one-way ANOVA with Tukey's multiple comparisons test with ****P<0.0001, ***P<0.001, **P<0.01, *P<0.05, and ns = not significant. See Table S7 for numerical values and additional statistics.

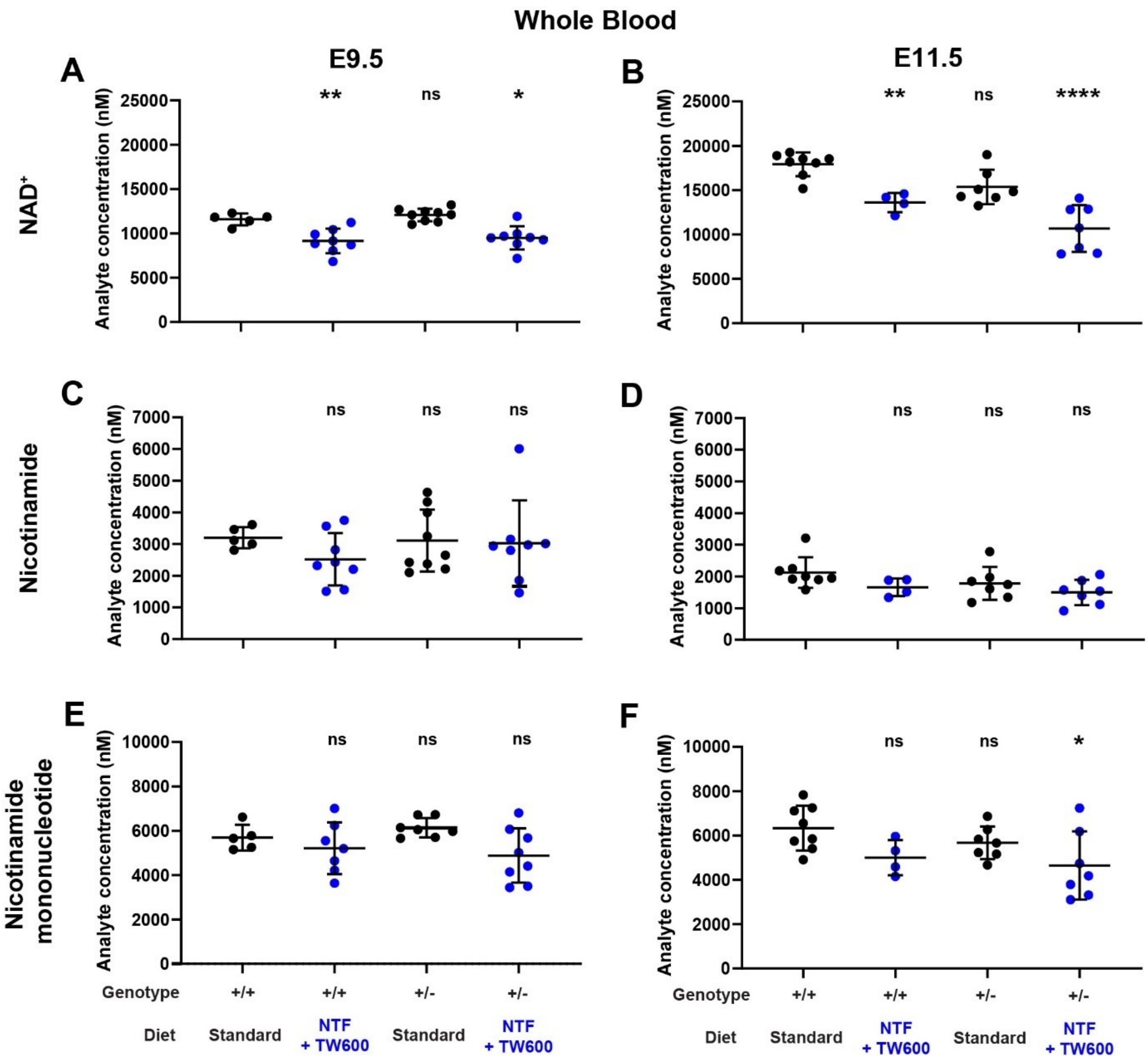

Fig. S11. NAD⁺, nicotinamide, and nicotinamide mononucleotide concentration in whole blood of pregnant wild-type and *Slc6a19*$^{+/-}$ mice, measured by UHPLC-MS/MS. Whole blood concentrations at E9.5(**A**, **C**, **E**) and at E11.5 (**B**, **D**, **F**). Dots represent metabolite concentrations in individual mice, and bars indicate the mean ± s.d.. The maternal *Slc6a19* genotype and diet are indicated at the bottom. Significance of differences in metabolite concentration relative to the pregnant wild-type standard diet group (first column) was assessed by one-way ANOVA with Tukey's multiple comparisons test with ****P<0.0001, **P<0.01, *P<0.05, and ns = not significant. See Table S7 for numerical values and additional statistics.

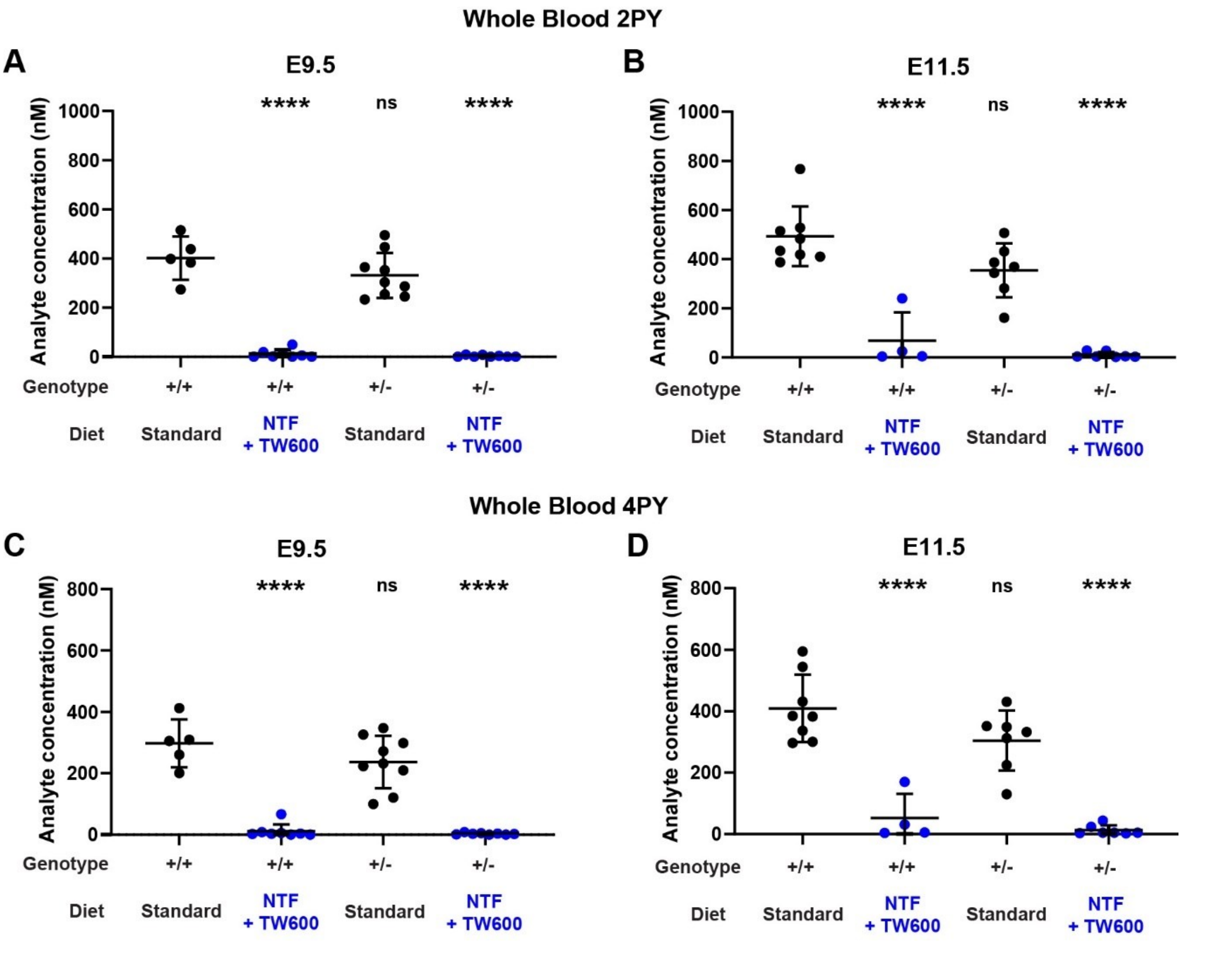

Fig. S12. 2PY and 4PY concentration in whole blood of pregnant wild-type and *Slc6a19*$^{+/-}$ mice, measured by UHPLC-MS/MS. Whole blood concentrations at E9.5 (**A**, **C**) and at E11.5 (**B**, **D**). Dots represent metabolite concentrations in individual mice, and bars indicate the mean ± s.d.. The maternal *Slc6a19* genotype and diet are indicated underneath each graph. Significance of differences in metabolite concentration relative to the pregnant wild-type standard diet group (first column) was assessed by one-way ANOVA with Tukey's multiple comparisons test with ****P<0.0001 and ns = not significant. See Table S7 for numerical values and additional statistics

Disease Models & Mechanisms • Supplementary information

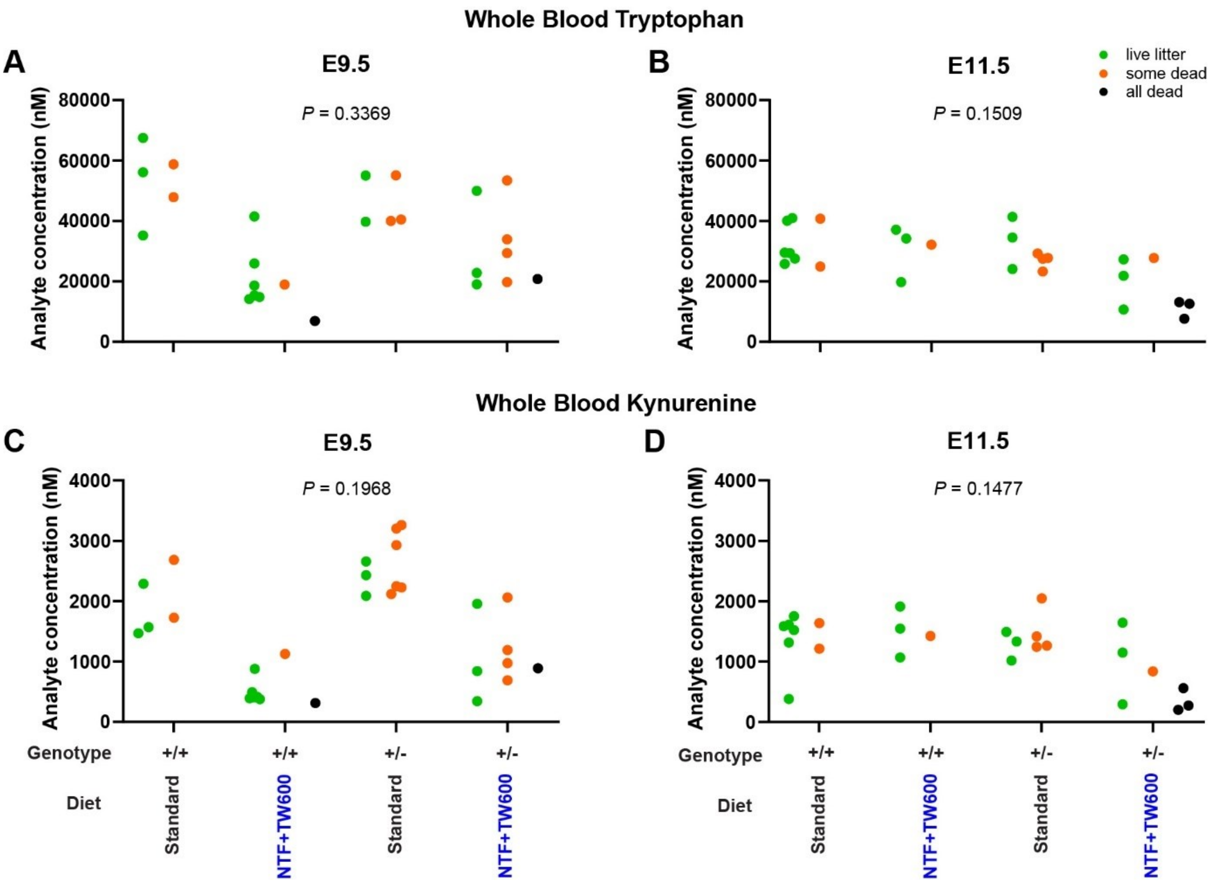

Fig. S13. Tryptophan and kynurenine concentrations in whole blood of pregnant wild-type and *Slc6a19*$^{+/-}$ mice and their correlation with the litter survival at the respective timepoints in pregnancy. Whole blood metabolite concentrations at E9.5 (**A**, **C**) and at E11.5 (**B**, **D**). Colours indicate the litter survival status per mother at dissection, with green: all embryos alive, orange: one or more embryos dead, black: all embryos dead. The maternal *Slc6a19* genotype and diet are indicated underneath each graph. Trends between litter survival and maternal metabolite levels were assessed by two-way ANOVA, comparing the 3 different litter outcomes across all 4 different maternal diet/genotype conditions. *P* values are indicated within the graphs. This dataset is related to Table S7 and Fig. S10.

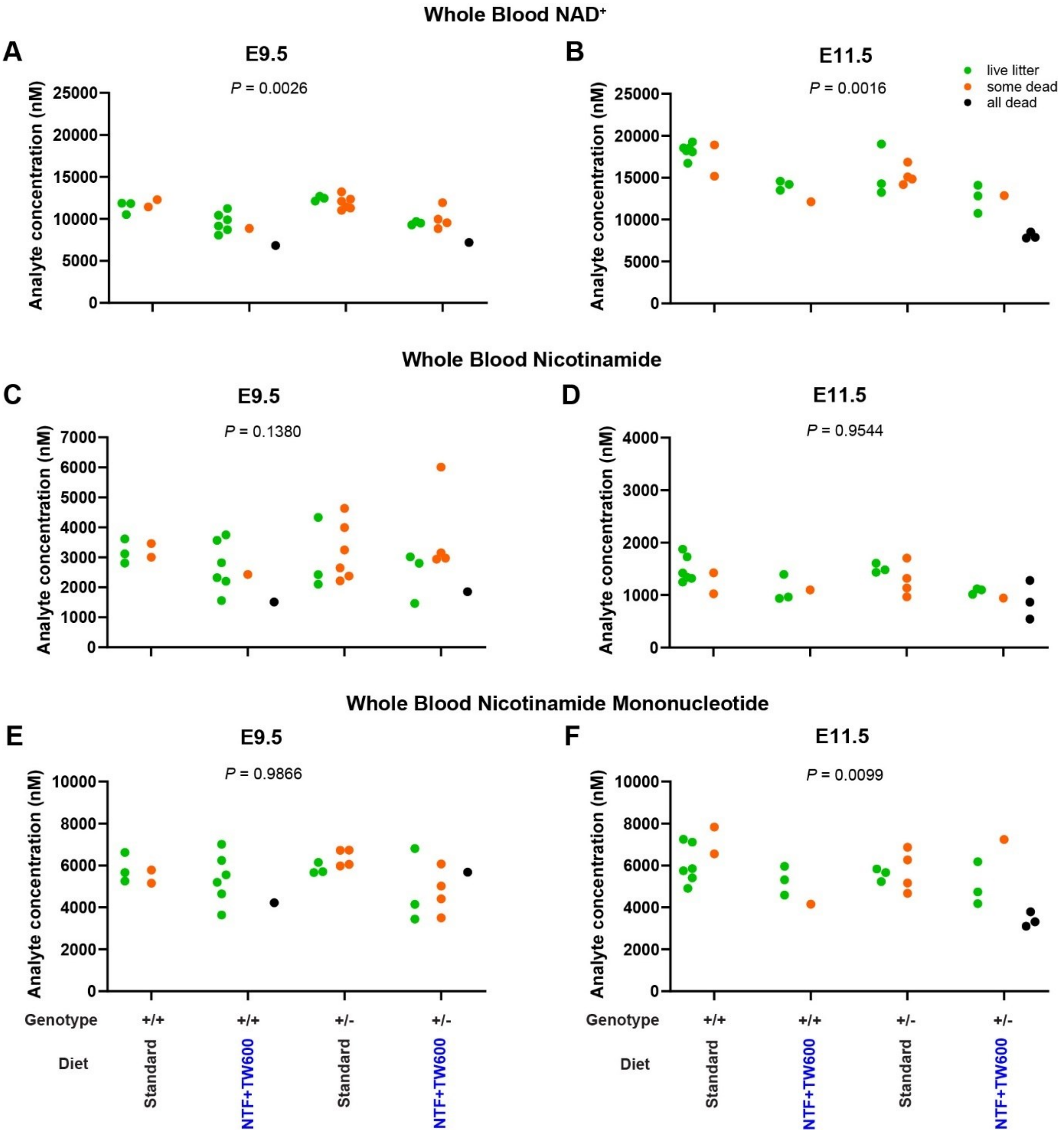

Fig. S14. NAD⁺, nicotinamide, and nicotinamide mononucleotide concentrations in whole blood of pregnant wild-type and *Slc6a19*$^{+/-}$ mice and their correlation with the litter survival at the respective timepoints in pregnancy. Whole blood metabolite concentrations at E9.5 (**A**, **C**, **E**); at E11.5 (**B**, **D**, **F**). Colours indicate the litter survival status per mother at dissection, with green: all embryos alive, orange: some embryos dead, black: all embryos dead. The maternal *Slc6a19* genotype and diet are indicated underneath each graph. Trends between litter survival and maternal metabolite levels were assessed by two-way ANOVA, comparing the 3 different litter outcomes across all 4 different maternal diet/genotype conditions. *P* values are indicated within the graphs. This dataset is related to Table S7 and Fig. S11.

Disease Models & Mechanisms • Supplementary information

Table S1. Overview of the mouse diets and their abbreviations used throughout the manuscript

Diet	Vitamin B3 in feed (mg/kg)	TRP in feed (mg/kg)	NA in water (mg/L)	TRP in water (mg/L)	TRP (% Standard diet)[2]	NAD precursors (% Standard diet)[2]	Dietary TRP (mg/d)[1]	Dietary NAD precursors (µg/d)[1]
Standard	31.4	2700	0	0			10.53	298.0
NTF+TW500	1.4	0	0	500	29.4	19.2	3.10	57.1
NTF+TW600	1.4	0	0	600	35.3	22.7	3.72	67.5
NTF+TW800	1.4	0	0	800	47.1	29.6	4.96	88.1
NTF+TW500+NW15	1.4	0	15.0	500	29.4	50.4	3.10	150.1
Breeding	~90	3700	0	0	137.0	198.7	14.43	~592

[1]Amounts of NAD precursors (=niacin equivalents) and tryptophan per day are based on an average consumption of 3.9 g food and 6.2 mL water per day and on the approximation that 60 mg of dietary tryptophan is equivalent to 1 mg of vitamin B3 for the conversion to NAD (Bachmanov et al., 2002; Goldsmith, 1958).
[2]The percentages refer to the NAD precursor and tryptophan supply, respectively, relative to the standard diet (first row). TRP, tryptophan; NA, nicotinic acid; NTF, NAD precursor vitamin-depleted and tryptophan-free feed; TW, tryptophan-supplemented water; NW, nicotinic acid-supplemented water.

Table S2. Statistical evaluation of embryo outcomes at E18.5, E11.5, and E9.5 with different parental *Slc6a19* genotypes and maternal diets during pregnancy

Row	Stage	M	P	Diet	Compared conditions[1]	*P* (Alive/ Dead)	*P* (Normal/ Malformed)
**a**	E18.5	+/+	+/+	Standard[2]	a,b,c	0.2931	<0.0001
**b**	E18.5	+/+	+/+	NTF+TW600[2]	b,f	0.0363	ns
**c**	E18.5	+/+	+/+	NTF+TW500[2]	c,g	<0.0001	<0.0001
**d**	E18.5	+/−	+/−	Standard	d,e,f,g	<0.0001	0.0104
**e**	E18.5	+/−	+/−	NTF+TW800	a,d	ns	ns
**f**	E18.5	+/−	+/−	NTF+TW600			
**g**	E18.5	+/−	+/−	NTF+TW500	g,h	<0.0001	ns
**h**	E18.5	+/−	+/−	NTF+TW500+NW15			
**i**	E11.5	+/+	+/+	Standard[2]	i,k	>0.9999	0.0662
**j**	E11.5	+/+	+/+	NTF+TW600[2]	j,l	0.0009	>0.9999
**k**	E11.5	+/−	+/−	Standard			
**l**	E11.5	+/−	+/−	NTF+TW600			
**m**	E9.5	+/+	+/+	Standard[2]	m,o	0.2593	0.4437
**n**	E9.5	+/+	+/+	NTF+TW600[2]	n,p	0.0092	0.0038
**o**	E9.5	+/−	+/−	Standard			
**p**	E9.5	+/−	+/−	NTF+TW600			

[1]Lowercase letters indicate which rows were compared. The rows also match the rows in Table 1 in the main manuscript. *P* values were calculated by two-sided Fisher's exact test (with Freeman-Halton extension) when comparing 2 or 3 groups, or χ^2 test when comparing multiple groups.
[2]Part of this dataset is from a previous study (Cuny et al., 2020).
M, maternal *Slc6a19* genotype; P, paternal *Slc6a19* genotype.
For the numbers and percentages of embryos for each dietary condition, see Table 1.

Table S3. Embryo weights at E18.5 with different *Slc6a19* genotypes and maternal dietary treatments during pregnancy

Row	M	P	Diet	Embryo weight (g ± s.d.)	n	*P* (one-way ANOVA)
a	+/+	+/+	Standard	1.12 ± 0.07	90	0.6187
b	+/+	+/+	NTF+TW600	1.07 ± 0.12	92	0.9944
c	+/+	+/+	NTF+TW500	0.98 ± 0.11	65	0.0001
d	+/−	+/−	Standard	1.08 ± 0.10	37	
e	+/−	+/−	NTF+TW500+NW15	1.01 ± 0.07	33	0.0791
f	+/−	+/−	NTF+TW800	0.97 ± 0.12	36	0.0002
g	+/−	+/−	NTF+TW600	0.94 ± 0.14	71	<0.0001
h	+/−	+/−	NTF+TW500	0.93 ± 0.13	17	<0.0001

P values were calculated by one-way ANOVA followed by Tukey's multiple comparisons test. The *P* values comparing the indicated treatment groups with the pregnant *Slc6a19*$^{+/-}$ on standard diet control group (row d) are shown. Additional comparisons: Effect of maternal *Slc6a19* genotype with NTF+TW600 diet (comparing rows b with g): *P*<0.0001. Effect of maternal *Slc6a19* genotype plus TRP restriction and vitamin B3 supplementation (comparing rows a with e): *P*<0.0001. M, maternal *Slc6a19* genotype; P, paternal *Slc6a19* genotype.

Table S4. Summary of all types and incidence of congenital malformations observed at E18.5.

			Standard[1]	NTF+TW600[1]	NTF+TW500[1]	Standard	NTF+TW800	NTF+TW600	NTF+TW500	NTF+TW500+ NW15
		M	+/+	+/+	+/+	+/-	+/-	+/-	+/-	+/-
**Location**	**Malformation**	P	+/+	+/+	+/+	+/-	+/-	+/-	+/-	+/-
Heart	*Total*		*3/90 (3%)*	*5/92 (5%)*	*16/65 (25%)*	*1/37 (1%)*	*6/36 (17%)*	*6/71 (8%)*	*1/17 (6%)*	-
	Bicuspid aortic valve (BAV)		2 (2%)	1 (1%)	5 (8%)	-	2 (6%)	5 (7%)	-	-
	Membranous ventricular septal defect (VSD)		1 (1%)	2 (2%)	9 (14%)	-	1 (3%)	-	1 (6%)	-
	Muscular ventricular septal defect (mVSD)		-	2 (2%)	4 (6%)	1 (3%)	3 (8%)	1 (1%)	-	-
	Overriding aorta (OA)		-	1 (1%)	5 (8%)	-	1 (3%)	-	1 (6%)	-
	Persistent truncus arteriosus (PTA)		-	-	2 (3%)	-	-	-	-	-
	Double outlet right ventricle (DORV)		-	-	2 (3%)	-	-	-	-	-
Vertebrae and ribs[2]	*Total*		*5/90 (6%)*	*11/92 (12%)*	*34/65 (52%)*	-	*3/36 (8%)*	*12/71 (17%)*	*3/17 (18%)*	*1/33 (3%)*
	Cervical vertebrae		4 (4%)	10 (11%)	27 (42%)	-	3 (8%)	10 (14%)	3 (18%)	1 (3%)
	Thoracic vertebrae		1 (1%)	1 (1%)	9 (14%)	-	1 (3%)	3 (4%)	1 (6%)	-
	Lumbar vertebrae		-	2 (2%)	6 (9%)	-	-	2 (3%)	2 (12%)	-
	Sacral vertebrae		-	3 (3%)	4 (6%)	-	-	-	1 (6%)	-
	Rib fusions		-	-	4 (6%)	-	-	-	-	-
	Extra pair of ribs		-	-	-	-	-	-	-	-
	Missing or underdeveloped ribs		-	-	1 (2%)	-	-	-	-	-
Kidneys[3]	*Total*		*1/90 (1%)*	*5/92 (5%)*	*21/65 (32%)*	*1/37 (1%)*	-	*8/71 (11%)*	*2/17 (12%)*	-
	Hypoplasia or agenesis		-	1 (1%)	19 (29%)	-	-	8 (11%)	2 (12%)	-
	Dysmorphic (duplex kidney, hydronephrosis)		1 (1%)	4 (4%)	2 (3%)	1 (3%)	-	-	-	-
Tail[4]	*Total*		-	*1/92 (1%)*	*27/65 (42%)*	-	-	*1/71 (1%)*	*1/17 (6%)*	-
	Caudal agenesis		-	1 (1%)	27 (42%)	-	-	1 (1%)	1 (6%)	-
Limbs	*Total*		-	-	*7/65 (11%)*	-	-	-	*1/17 (6%)*	-
	Talipes		-	-	5 (8%)	-	-	-	1 (6%)	-
	Short limbs		-	-	2 (3%)	-	-	-	-	-
Digits[5]	*Total*		-	*6/92 (7%)*	*36/65 (55%)*	-	-	*1/71 (1%)*	*2/17 (12%)*	-

	Polydactyly	-	6 (7%)	33 (51%)	-	-	-	2 (12%)	-
	Oligodactyly	-	-	2 (3%)	-	-	1 (1%)	-	-
	Syndactyly	-	-	2 (3%)	-	-	1 (1%)	-	-
Palate	*Total*	-	-	*15/65 (23%)*	-	-	*8/71 (11%)*	*1/17 (6%)*	-
	Cleft palate	-	-	15 (23%)	-	-	8 (11%)	1 (6%)	-
Eyes	*Total*	-	*11/92 (12%)*	*30/65 (46%)*	-	-	*9/71 (13%)*	*2/17 (12%)*	-
	Microphthalmia	-	10 (11%)	30 (46%)	-	-	7 (10%)	2 (12%)	-
	Coloboma	-	3 (3%)	-	-	-	3 (4%)	-	-
Abdominal wall	*Total*	-	*1/92 (1%)*	*9/65 (14%)*	-	-	*5/71 (7%)*	*1/17 (6%)*	-
	Omphalocele or Gastroschisis	-	1 (1%)	9 (14%)	-	-	5 (7%)	1 (6%)	-
Neural tube	*Total*	-	*2/92 (2%)*	*1/65 (2%)*	-	-	*1/71 (1%)*	*1/17 (6%)*	-
	Exencephaly	-	2 (2%)	1 (2%)	-	-	1 (1%)	1 (6%)	-

For representative images of several commonly occurring malformations, see Fig. S1. Uppercase letters M and P designate maternal and paternal *Slc6a19* genotype, respectively. Percentages summarise defects observed in live embryos only. Malformation "Total" refers to the total number of affected embryos for the given "Location", irrespective of the cumulative number of malformations.

[1]Dataset is from a previous study (Cuny et al., 2020).
[2]C1 and C2 were occasionally removed during dissection. Therefore, isolated defects in the vertebrae C1 and/or C2 were not counted. Ossification point abnormalities (e.g. flattened shape, less compaction, dumbbell shape, smaller than usual, two separate ossification points) were not counted, as they can result from delayed ossification and are not necessarily leading to a skeletal defect (Carney and Kimmel, 2007). Vertebral abnormalities observed and counted as congenital malformations included vertebral fusions, butterfly vertebrae, and hemivertebrae.
[3]Kidneys of wild-type E18.5 embryos developed under a normal unrestricted diet are consistently around 3 mm long measured from tip to tip (average 2.98 mm; range 2.75-3.375 mm; n=90) (Cuny et al., 2020). Kidneys ≤1.5 mm in length were deemed malformed and classified as hypoplastic.
[4]The skeletal staining procedure did not reliably allow visualisation of caudal vertebra defects. Therefore, tails were assessed at the whole embryo level. Short and curly tails not extending past the toes, even if the tail was stretched out, were classified as caudal agenesis.
[5]Polysyndactyly is counted as polydactyly and syndactyly.

Table S5. Statistical evaluation of embryo NAD levels at E9.5 and E11.5 with different *Slc6a19* genotypes and maternal dietary treatments during pregnancy

						one-way ANOVA	Two-way ANOVA		
Row	Stage	M	P	Diet	Compared conditions[1]	*P*	*P* (diet)	*P* (genotype)	*P* (interaction)
**a**	E9.5	+/+	+/+	Standard	a,c	0.1937	-	-	-
**b**	E9.5	+/+	+/+	NTF+TW600	b,d	0.9984	-	-	-
**c**	E9.5	+/−	+/−	Standard	c,d	<0.0001	<0.0001	0.6426	0.0353
**d**	E9.5	+/−	+/−	NTF+TW600					
**e**	E11.5	+/+	+/+	Standard[2]	e,g	0.0788	-	-	-
**f**	E11.5	+/+	+/+	NTF+TW600[2]	f,h	0.0991	-	-	-
**g**	E11.5	+/−	+/−	Standard	g,h	<0.0001	<0.0001	0.4469	0.9441
**h**	E11.5	+/−	+/−	NTF+TW600					

[1]Lowercase letters indicate which rows were compared. Comparisons between maternal wild-type and *Slc6a19*$^{+/-}$ groups were done by one-way ANOVA with Tukey's multiple comparisons test. To assess the effects of diet, embryonic *Slc6a19* genotype, and their interaction, both maternal *Slc6a19*$^{+/-}$ groups (standard diet and NTF+TW600) were compared by two-way ANOVA, taking into account the 3 different embryonic *Slc6a19* genotypes from *Slc6a19*$^{+/-}$ x *Slc6a19*$^{+/-}$ matings.

[2]Part of this dataset is from a previous study (Cuny et al., 2020).

M, maternal *Slc6a19* genotype; P, paternal *Slc6a19* genotype.

For the embryo numbers and NAD levels for each dietary condition, see Table 2.

Table S6. Decidua NAD levels at E7.5 in mice with different *Slc6a19* genotypes and maternal dietary treatments during pregnancy

Row	M	P	Embryo	Diet	NAD (nmol/mg protein ± s.d.)	n	*P* (one-way ANOVA)
a	+/+	+/+	+/+	Standard	7.10 ± 1.08	29	
b	+/+	+/+	+/+	NTF+TW600	6.29 ± 1.25	20	0.1344
c	+/−	+/−	all	Standard	6.04 ± 1.63	20	0.0263
d	+/−	+/−	all	NTF+TW600	6.14 ± 1.09	16	0.0810

P values were calculated by one-way ANOVA followed by Tukey's multiple comparisons test. The *P* values comparing the indicated treatment groups with the pregnant wild-type on standard diet control group (row a) are shown.

Table S7. Maternal whole blood NAD metabolite concentrations of pregnant mice at E9.5 and E11.5 with different *Slc6a19* genotypes and maternal dietary treatments during pregnancy, as measured by UHPLC-MS/MS

Stage	M	Diet	TRP (µM)	KYN (µM)	NAD (nM)	NAM (µM)	NMN (µM)	2PY (nM)	4PY (nM)	KYN:TRP (nM:µM)[1]
E9.5	+/+	Standard	53.1 ± 12	1.95 ± 0.52	11.6 ± 0.67	3.20 ± 0.33	5.69 ± 0.58	402 ± 88	298 ± 78	38.5 ± 13
E9.5	+/−	Standard	46.1 ± 8.2	2.57 ± 0.46	12.1 ± 0.71	3.11 ± 0.98	6.14 ± 0.44	331 ± 92	237 ± 85	52.0 ± 8.5
P			0.7736	0.1103	0.8510	0.9983	0.8563	0.2012	0.2519	0.3867
E9.5	+/+	NTF+TW600	19.5 ± 10	0.550 ± 0.29	9.15 ± 1.4	2.52 ± 0.83	5.21 ± 1.2	11.1 ± 17	11.2 ± 22	33.2 ± 17
E9.5	+/−	NTF+TW600	31.1 ± 14	1.12 ± 0.60	9.49 ± 1.3	3.02 ± 1.4	4.88 ± 1.2	2.55 ± 3.8	3.06 ± 2.9	35.7 ± 11
P			0.2144	0.1049	0.9225	0.7425	0.9078	0.9926	0.9919	0.9796
E11.5	+/+	Standard	32.4 ± 7.0	1.38 ± 0.44	17.9 ± 1.3	2.12 ± 0.48	6.33 ± 1.0	493 ± 122	409 ± 110	44.1 ± 18
E11.5	+/−	Standard	29.7 ± 6.3	1.40 ± 0.32	15.3 ± 2.0	1.78 ± 0.52	5.67 ± 0.74	354 ± 110	304 ± 98	49.2 ± 15
P			0.8904	0.9994	0.0739	0.4756	0.6531	0.0595	0.1165	0.9206
E11.5	+/+	NTF+TW600	30.8 ± 7.6	1.49 ± 0.35	13.6 ± 1.1	1.66 ± 0.28	5.00 ± 0.80	68.3 ± 115	52.3 ± 79	49.0 ± 7.0
E11.5	+/−	NTF+TW600	17.3 ± 8.3	0.709 ± 0.54	10.7 ± 2.6	1.50 ± 0.40	4.65 ± 1.5	10.6 ± 12	12.2 ± 16	38.0 ± 15
P			0.0334	0.0393	0.0994	0.9379	0.9554	0.7910	0.8786	0.6783

Data is from 4-9 mice per condition at each gestational stage and shown as mean ± s.d.. *P* values were calculated by one-way ANOVA followed by Tukey's multiple comparisons test and values for comparisons between wild-type and *Slc6a19*$^{+/-}$ mothers at the indicated gestational stage and diet are shown within the table.

In addition, significance of difference to the wild-type standard diet control group was evaluated. At E9.5, for wild-type NTF+TW600, the following values were significantly different: TRP *P*=0.0002, KYN *P*=0.0001, NAD$^+$ *P*=0.0032, 2PY *P*<0.0001, and 4PY *P*<0.0001. For *Slc6a19*$^{+/-}$ standard diet, these were: none. For *Slc6a19*$^{+/-}$ NTF +TW600, these were: TRP *P*=0.0148, KYN *P*=0.0251, NAD$^+$ *P*=0.0124, 2PY *P*<0.0001, and 4PY *P*<0.0001.

At E11.5, for wild-type NTF+TW600, the following values were significantly different to the wild-type standard diet control group: NAD$^+$ *P*=0.0066, 2PY *P*<0.0001, 4PY *P*<0.0001. For *Slc6a19*$^{+/-}$ standard diet, these were: none. For *Slc6a19*$^{+/-}$ NTF+TW600, these were: TRP *P*=0.0031, KYN *P*=0.0307, NAD *P*<0.0001, NMN *P*=0.0338, 2PY *P*<0.0001, 4PY *P*<0.0001.

[1]KYN:TRP ratio was calculated for each sample individually, then the mean ± s.d. calculated from these values.

M, maternal *Slc6a19* genotype; TRP, tryptophan; KYN, kynurenine; NAM, nicotinamide; NMN, nicotinamide mononucleotide; 2PY, N-methyl-2-pyridone-5-carboxamide; 4PY, N-methyl-4-pyridone- 5-carboxamide.

Supplemental references

Bachmanov, A. A., Reed, D. R., Beauchamp, G. K. and Tordoff, M. G. (2002). Food intake, water intake, and drinking spout side preference of 28 mouse strains. *Behav Genet* **32**, 435-43.

Carney, E. W. and Kimmel, C. A. (2007). Interpretation of skeletal variations for human risk assessment: delayed ossification and wavy ribs. *Birth Defects Res B Dev Reprod Toxicol* **80**, 473-96.

Cuny, H., Rapadas, M., Gereis, J., Martin, E., Kirk, R. B., Shi, H. and Dunwoodie, S. L. (2020). NAD deficiency due to environmental factors or gene-environment interactions causes congenital malformations and miscarriage in mice. *Proc Natl Acad Sci U S A* **117**, 3738-3747.

Goldsmith, G. A. (1958). Niacin-tryptophan relationships in man and niacin requirement. *Am J Clin Nutr* **6**, 479-86.

Moreau, J. L., Artap, S. T., Shi, H., Chapman, G., Leone, G., Sparrow, D. B. and Dunwoodie, S. L. (2014). Cited2 is required in trophoblasts for correct placental capillary patterning. *Dev Biol* **392**, 62-79.

O'Reilly, V. C., Lopes Floro, K., Shi, H., Chapman, B. E., Preis, J. I., James, A. C., Chapman, G., Harvey, R. P., Johnson, R. S., Grieve, S. M. et al. (2014). Gene-environment interaction demonstrates the vulnerability of the embryonic heart. *Dev Biol* **391**, 99-110.
